# The Effects of Graded Levels of Calorie Restriction: XIII. Global Metabolomics Screen Reveals Graded Changes in Circulating Amino Acids, Vitamins, and Bile Acids in the Plasma of C57BL/6 Mice

**DOI:** 10.1093/gerona/gly058

**Published:** 2018-04-30

**Authors:** Cara L Green, Quinlyn A Soltow, Sharon E Mitchell, Davina Derous, Yingchun Wang, Luonan Chen, Jing-Dong J Han, Daniel E L Promislow, David Lusseau, Alex Douglas, Dean P Jones, John R Speakman

**Affiliations:** 1 Institute of Biological and Environmental Sciences, University of Aberdeen, UK; 2 Division of Pulmonary, Allergy and Critical Care Medicine, Clinical Biomarkers Laboratory, Department of Medicine, Emory University, Atlanta, Georgia; 3 State Key laboratory of Molecular Developmental Biology, Institute of Genetics and Developmental Biology, Chinese Academy of Sciences, Chaoyang, Beijing, China; 4 Key laboratory of Systems Biology, Innovation Center for Cell Signaling Network, Institute of Biochemistry and Cell Biology, China; 5 Key Laboratory of Computational Biology, Chinese Academy of Sciences-Max Planck Partner Institute for Computational Biology, Shanghai Institutes for Biological Sciences, Chinese Academy of Sciences, China; 6 Department of Pathology, Seattle; 7 Department of Biology, University of Washington, Seattle

**Keywords:** Metabolomics, Calorie restriction, Aging, Vitamin E, Amino acids, Bile acids

## Abstract

Calorie restriction (CR) remains the most robust intervention to extend life span and improve health span. Using a global mass spectrometry–based metabolomics approach, we identified metabolites that were significantly differentially expressed in the plasma of C57BL/6 mice, fed graded levels of calorie restriction (10% CR, 20% CR, 30% CR, and 40% CR) compared with mice fed ad libitum for 12 hours a day. The differential expression of metabolites increased with the severity of CR. Pathway analysis revealed that graded CR had an impact on vitamin E and vitamin B levels, branched chain amino acids, aromatic amino acids, and fatty acid pathways. The majority of amino acids correlated positively with fat-free mass and visceral fat mass, indicating a strong relationship with body composition and vitamin E metabolites correlated with stomach and colon size, which may allude to the beneficial effects of investing in gastrointestinal organs with CR. In addition, metabolites that showed a graded effect, such as the sphinganines, carnitines, and bile acids, match our previous study on liver, which suggests not only that CR remodels the metabolome in a way that promotes energy efficiency, but also that some changes are conserved across tissues.

Aging and age-associated diseases are the primary cause of mortality in developed countries and are on the rise in developing countries, where the rate of population aging is accelerating faster than the developed countries in the past (1–3). Consequently, we need to find innovative ways to sustain “healthy aging” to manage our aging populations. Calorie restriction (CR) can delay and reverse the effects of aging in a wide range of organisms including *Caernahabditis elegans*, *Drosophila melanogaster*, fish, rodents, nonhuman primates, and possibly even humans (4). In rodents, decreasing calorie intake from 0% to 65% is linearly associated with increasing life span (5). As the life span–extending effect of CR increases with higher levels of CR, a study design involving graded levels of CR is useful to identify potential biological pathways that may be important to the beneficial effects of CR on aging. Recently, the use of graded CR has become a popular approach (6–8).

Focusing on plasma provides a means to take an integrative view of changes across different organs. Plasma facilitates the detoxification and removal of waste products, maintains water and salt balance, and transports nutrients between organs. As plasma can be readily sampled, it is ideal for metabolomic biomarker discovery and has been used for many diseases including liver cirrhosis and liver carcinoma (9), breast cancer (10), and pulmonary arterial hypertension (11). Liquid chromatography–mass spectrometry provides sensitive detection of metabolites. Using a broad-spectrum metabolomic platform for untargeted analysis allows the detection of metabolites that may not have been previously associated with CR and aging. Previously, metabolomic analyses of the liver from mice on graded CR indicated that CR is associated with an increase in carnitines, phospholipids, and bile acids, indicating that under higher levels of CR, the liver becomes increasingly efficient at breaking down and metabolizing lipids (12).

Plasma metabolomic profiles show age-related changes in protein and vitamin levels (13). In particular, vitamin E exhibits a significant decrease in its several forms, with plasma γ-tocopherol and the platelet α-tocopherol, γ-tocopherol, and total tocopherol concentrations decreasing significantly with age (14). Global plasma metabolomics of mice at 30% CR have shown changes in amino acid profiles, lactate, cholesterol, and low-density lipoproteins after only 48 hours of 40% of CR in C57BL/6 mice (15).

In this study, we determined differential metabolite expression between a control group fed ad libitum during the 12 hours of darkness each 24-hour cycle (12AL), a control group fed ad libitum 24 hours a day (24AL), and CR groups (10% CR–40% CR). Our goal was to detect metabolites and pathway changes with CR. Looking at each individual CR group allowed us to detect nonlinear changes such as those that have a threshold level. We also aimed to determine whether any metabolites or pathways changed in a linear fashion with increasing restriction, as these changes may mirror the relationship between life span and CR.

## Experimental Procedures

### Experimental Design

Our dataset consisted of metabolomic data from technical duplicates of 46 individual plasma samples. Mice were allocated to six treatment groups: control (12AL: 12-hour ad libitum feeding during the hours of darkness only and 24AL: 24-hour ad libitum feeding) and 10% CR, 20% CR, 30% CR, and 40% CR (percentage of calories less than individual average food intakes measured over a 2-week baseline period) for 3 months (10CR, 20CR, 30CR, and 40CR, respectively). Sample size was eight for all groups except 24AL and 30CR where *n* = 7. We initiated CR at 20 weeks; this was done to avoid any effect of CR on development while retaining effectiveness of increasing life span.

### Animals

Five-month-old C57BL/6 male mice were used (16). All procedures were reviewed and approved by University of Aberdeen ethical approval committee and carried out under a Home Office issued license compliant with the Animals (Scientific Procedures) Act 1986. This strain is already known to live longer under CR (17). Mice were purchased from Charles River (Ormiston, UK). Free access to water was provided. More detailed information on study design can be found in Supplementary S1 and in the study by Mitchell and colleagues (16).

### Liquid Chromatography–Mass Spectrometry

Metabolite features were identified with dual chromatography–Fourier transform mass spectrometry (DC-FTMS) using electrospray ionization in the positive mode. Replicates were run at the same time, and samples were run in a randomized order. Samples were run in two batches. Methodology on the dual chromatography–liquid chromatography–mass spectrometry procedure can be found in ref. (18) and in Supplementary S2. A Thermo LTQ-FT mass spectrometer (linear ion trap with a Fourier Transform Ion Cyclotron Resonance [FT-ICR] MS detector, Thermo Fischer, San Diego, CA) was set to collect data between 85 and 850 *m*/*z* (mass–charge ratio).

### Metabolomic Preprocessing

As metabolites, even within samples, show a significant amount of stochastic variation, it is necessary to filter out “noisy,” uninformative metabolites. First, metabolites were normalized using a log 2 transformation. Second, only metabolites with a signal-to-noise ratio (SNR_*i*_ = mean/sample *SD*) ≥ 15 were retained for analysis. Third, metabolites that were missing from 15% or more of all samples were excluded.

### Statistical Modeling of Differential Metabolite Expression

To detect significantly differentially expressed metabolites between treatment groups, an empirical Bayes moderated linear model was fitted to each metabolite (19). The empirical Bayes approach shrinks the estimated sample variances by borrowing information from across metabolites. Fold changes were estimated using contrasts between 12AL and each level of CR (10%, 20%, 30%, and 40%), plus the 24AL group. *p* Values for each comparison were adjusted using the Benjamini-Hochberg (BH) procedure using a false discovery rate of 5% (20). We used the Devium package to perform orthogonal - partial least squares - discriminant analysis (O-PLS-DA), to complete the validation steps and retrieve metabolite loadings, and to produce the biplot and scores plots (21) (see Supplementary S3 for more information).

### Putative Identification of Metabolites

Metabolites were identified using two approaches. First, metabolites were assigned using xMSannotator by the Human Metabolome Database (HMDB), Kyoto Encyclopaedia of Genes and Genomes (KEGG), and LipidMaps (22). Second, the metabolite enrichment program *mummichog* (23) was used to assign putative metabolite matches based on pathways that are enriched within the metabolite set using a reference model derived from integration of KEGG, biochemically, genetically, and genomically structured (BiGG) and Edinburgh Human Metabolic Network (EHMN) databases.

### Biological Pathway Analysis

Pathway analysis was conducted using *mummichog* and ingenuity pathway analysis (IPA). For analysis in *mummichog*, features with unadjusted *p* ≤ .05 generated by the empirical Bayes linear model for each comparison with 12AL were utilized, along with their corresponding log fold changes and retention times. Metabolites identified in both *mummichog* and *xMSannotator*, in addition to their unadjusted *p* values and fold-changes were entered into IPA for analysis. Only metabolites with an ID from either KEGG or HMDB were used (see Supplementary S4 for more information).

### Correlations With Physiological Parameters

Fat-free mass, adiposity, stomach, and colon weight (16); circulating leptin and insulin, catalase, and derivatives of reactive oxidative metabolites (24); and hypothalamic transcript levels for cholinergic receptors (25) were correlated with log 2 transformed, normalized metabolite intensities for each individual mouse using Pearson’s correlation. Associated *p* values for each correlation were adjusted using the BH procedure using a false discovery rate of 5%.

## Results

### Characterization of Metabolomic Plasma Profiles Using Mass Spectrometry

**Figure 1.**
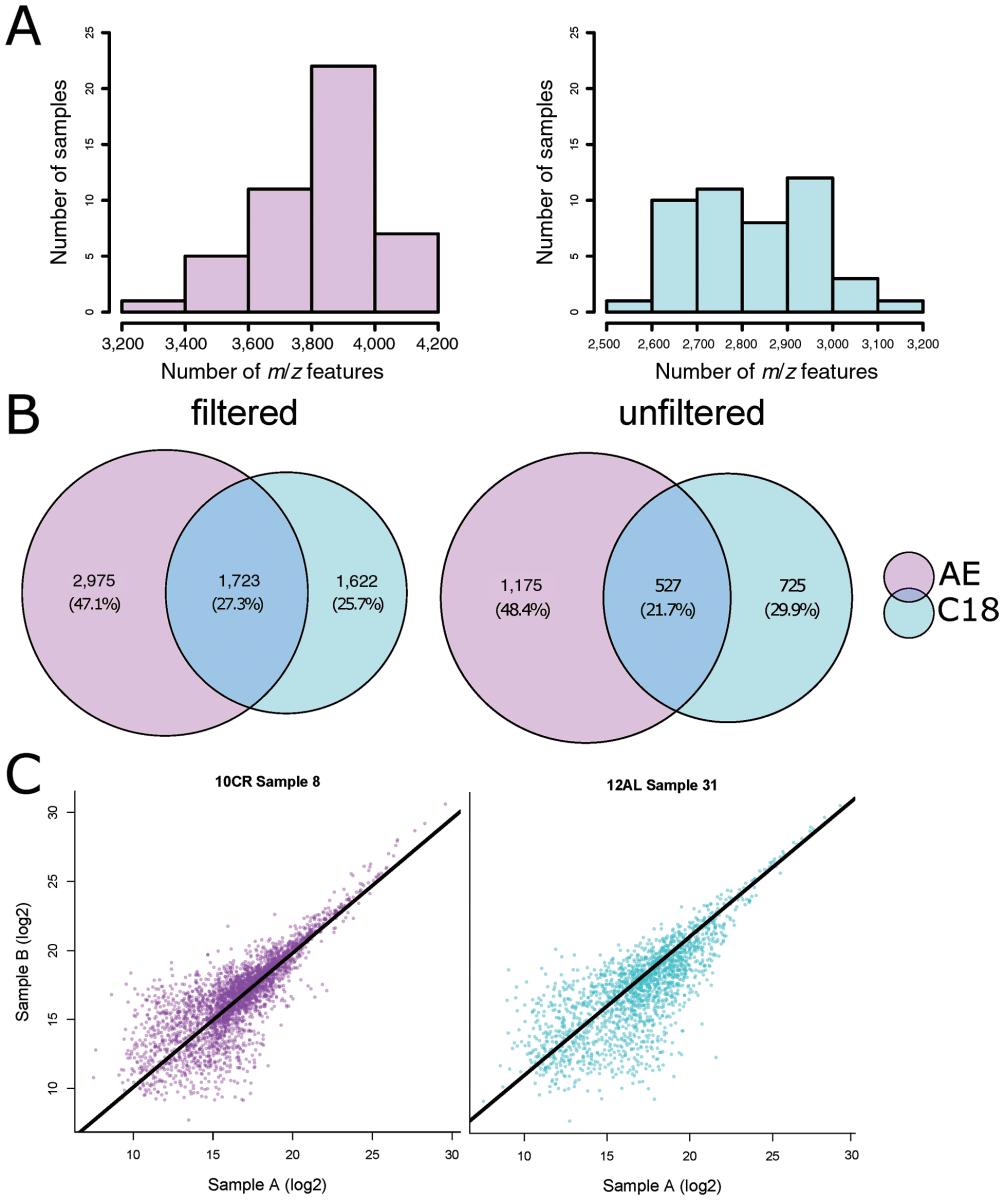
(**A**) Histogram showing distribution of features detected in 46 mice samples. AE: anion exchange column; C18: reverse phase column. (**B**) Venn diagram metabolites detected, overlap between columns. (**C**) Correlation of technical replicates in AE and C18 columns. Correlations were applied to all filtered metabolites, intensities were log_2_ transformed. AE: standardized major axis (SMA) gradient = 0.973, *R* = .809, *p* < .001. C18: SMA gradient = 0.980, *R* = .738, *p* < .001.

CR was applied to male mice at 20 weeks of age for 12 weeks. Control mice were allowed ad libitum access to food for 12 hours (time-restricted feeding, 12AL) and 24 hours a day (24AL), and the four CR groups were given 10%, 20%, 30% and 40% restriction from their baseline intakes. 12AL was used as the control group for all further analyses to remove any potential “time since last meal effect” of 24AL mice, as all mice had been food deprived for at least 7.5 hours before culling. We used DC-FTMS to detect metabolomic features, characterized by unique retention times and *m*/*z* ratios. We found that the anion exchange (AE) method detected 4,698 *m*/*z* features and the C18 or reverse phase method detected 3,345 *m*/*z* features. Although the number of *m*/*z* features across individuals differed between the AE and C18 columns, the median *m*/*z* values for the methods were similar (AE median *m*/*z* = 427.60; C18 median *m*/*z* = 437.20 (Figure 1A). To determine the contribution of each column to the overall profile of metabolites detected, *m*/*z* values were matched between the AE and C18 feature tables (Figure 1B). A total of 8,043 *m*/*z* features were found in the overall profile, 27% of which were detected in both the AE and C18. The majority of the *m*/*z* features were unique to the AE analysis (47%). The C18 analysis added 1,622 *m*/*z* features that were not detected by the AE separation, approximately 26%. The *m*/*z* values were filtered based on their replication across individuals and signal-to-noise ratio. Postfiltering, a total of 2,954 *m*/*z* features were present, 1.702 from AE and 1,252 from C18. Technical duplicates showed strong correlations across all samples (AE: *R* = .64–.81, adjusted *p* < .001, C18: *R* = .63–.87 adjusted *p* < .001; Figure 1C). Of the filtered metabolites in the AE and C18 columns, 755 and 491 features, respectively, were significantly differentially expressed (*F* statistic *p* < .05). As the level of restriction increased, the number of significantly differentially expressed features also rose in both AE and C18 columns (Supplementary Tables 1 and 2). Subsequently, features from both columns were combined into a master list for subsequent analyses. Metabolites were identified using *mummichog* and *xMSannotator*, using KEGG, HMDB, LipidMaps, and T3DB. Of the 2,954 *m*/*z* values, we were able to putatively assign IDs to 713 metabolites.

### Discriminant Analysis

**Figure 2.**
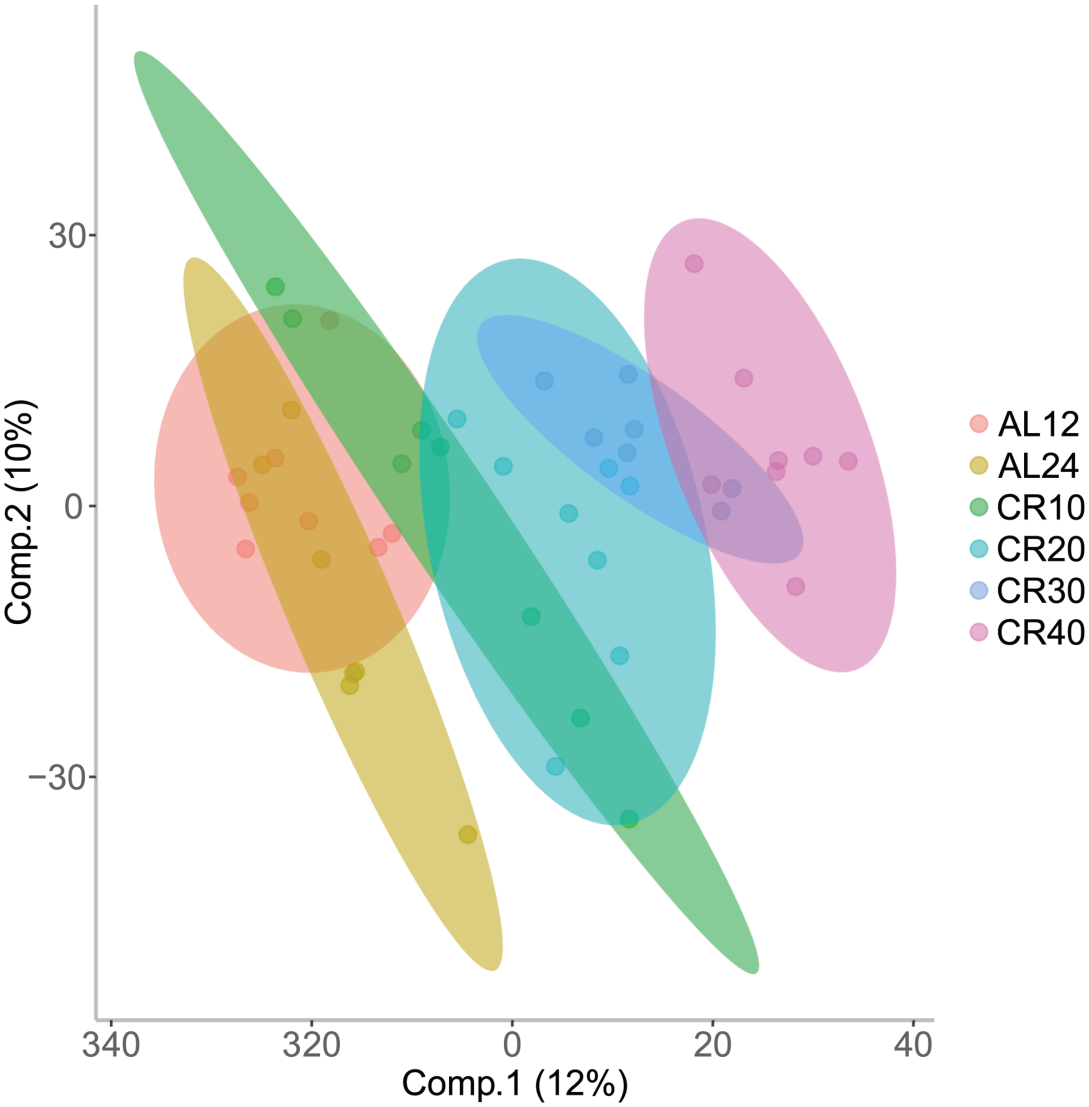
Orthogonal partial least square discriminate analysis (O-PLS-DA) demonstrates the differentiation effect of diet in metabolomic profiles. Loadings plot shows significant separation among samples on the basis of the model quality parameters: RX^2^, *Q*^2^, and RMSEP.

**Figure 3.**
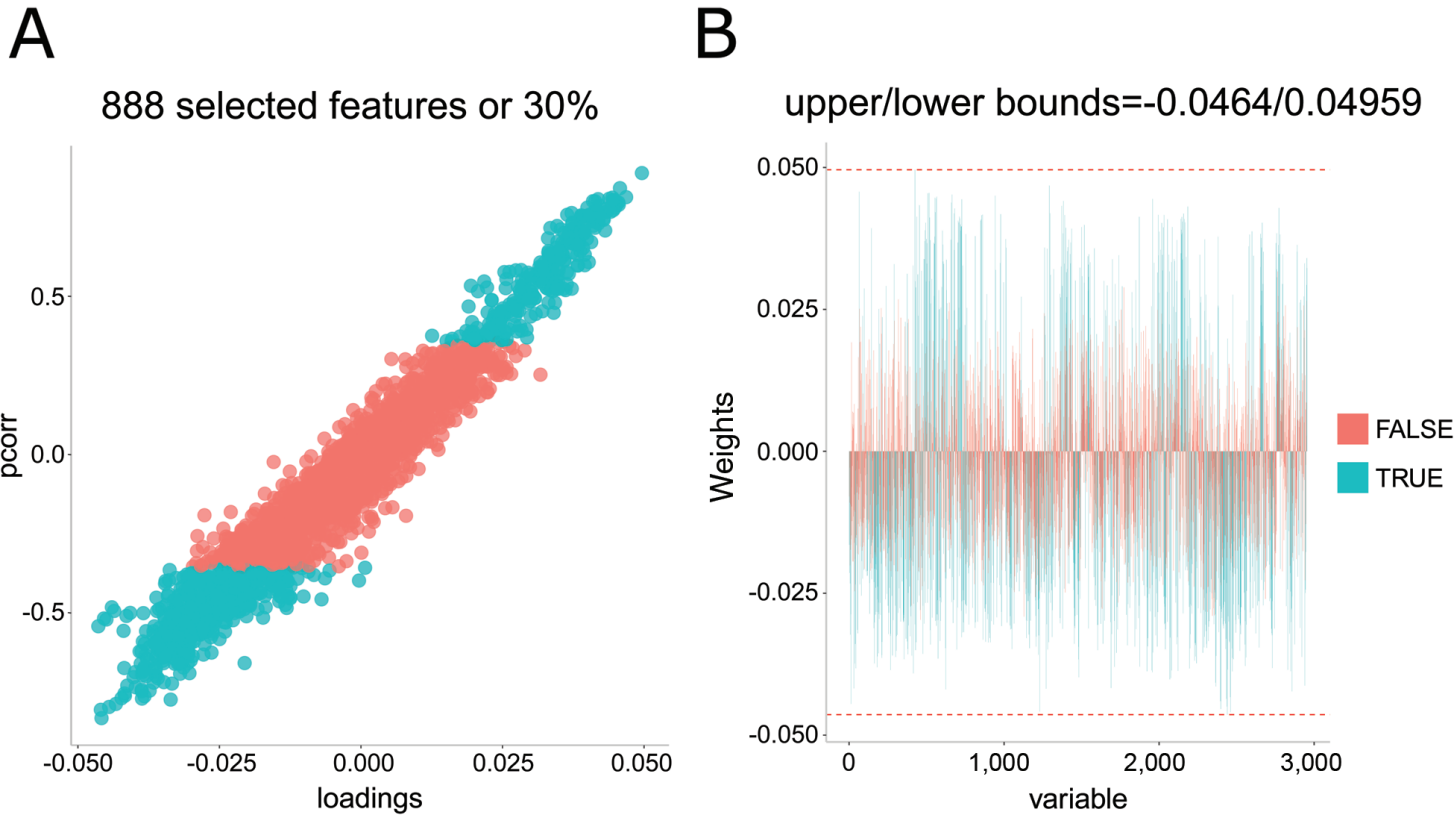
(**A**) S-plot indicating in turquoise metabolites that were significantly contributing to differences among feeding groups (false discovery rate cutoff *p* < .05). (**B**) Bar plot showing the weights of those metabolites that were significant (turquoise).

We performed an O-PLS-DA analysis on all identified and nonidentified filtered features (*n* = 2,954). The model indicated that around 60% of the variance in the features could be explained by the dietary treatment (single-sample *t* test between model parameters and permuted parameters, *n* = 1,000, *p* < .001, RX^2^ = 58.24, *Q*^2^ = 0.8038, RMSEP = 0.7518; Figure 2). Loadings from the O-PLS-DA model determined features that contributed to the separation of groups (Figure 3). Of 2,954 *m*/*z* features, 888 features contributed significantly to discrimination between treatment groups, using a false discovery rate cutoff *p* < .05. Of these, we were able to identify 224 (Supplementary Table 3). The model indicated that several vitamins, including B2, B5, and numerous vitamin E derivatives, carnitine and taurine metabolites, amino acids, sphinganine, and sphinganine-1-phosphate were important in discriminating the different CR groups.

Normalized metabolite intensities were correlated across each individual mouse between the plasma and the liver. Metabolites that correlated positively between the plasma and the liver included creatine, O-acetylcarnitine and O-propanoylcarnitine, l-methionine, and 15-keto-prostaglandin E2. Plasma l-arginine was significantly negatively correlated with intensities of the same metabolite in the liver.

### Pathway Analysis

For 10CR, 20CR, 30CR, and 40CR, we carried out a metabolite set enrichment analysis using *mummichog* and IPA. *Mummichog* identifies metabolites and pathways based on *m*/*z* values, fold changes, *p* values, and retention times (Supplementary Table 4). IPA identifies pathways using putatively identified metabolites through *mummichog* and *xMSannotator* with their associated fold changes and *p* values (*n* = 713). Of these IDs, 321 were recognized in the IPA database and were analyzed.

### Changes Across Several Amino Acids

Mummichog analysis showed that features in several amino acid pathways altered with CR, in particular at 40CR, although there were modifications at all levels of restriction. The majority of features in amino acids were decreased (Supplementary Table 5). In particular, decreases in features belonging to the aromatic amino acid pathways such as tyrosine and tryptophan were observed. Moreover, branched chain amino acid (BCAA) pathways in addition to aspartate, asparagine, and lysine also appeared decreased. IPA analysis indicated that at 40CR tyrosine biosynthesis (*p* < .01), phenylalanine degradation (*p* < .01), and valine degradation (*p* = .04) were also significantly decreased. At 10CR, a different set of pathways appeared altered in IPA including proline biosynthesis (*p* < .01) and leucine degradation (*p* = .01), which showed a decrease relative to 12AL.

### Vitamins Classes Increased by CR

**Figure 4.**
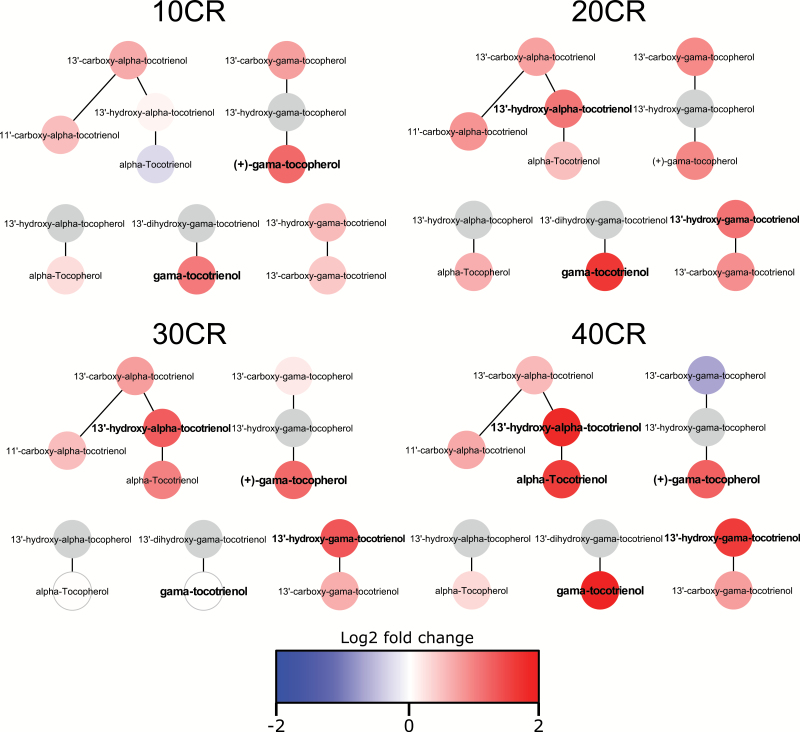
Vitamin E metabolism pathway at 10% CR–40% CR. Bold indicates metabolite significantly differentially expressed between CR group and 12AL. Gray indicates metabolite not detected in sample.

Components of the vitamin E metabolism pathway showed significant increases at 20CR, 30CR, and 40CR (*p* < .01 in all cases, Figure 4). At 40CR, six metabolites in the vitamin D3 metabolism pathway and two metabolites in the cofactor vitamin B6 metabolism pathway increased significantly relative to 12AL (*p* < .01 and *p* = .01, respectively). The vitamin D3 pathway was also significantly higher at 20CR with three features significantly increased (*p* = .03) compared with 12AL.

### Fatty Acid Pathways Were Altered With CR

As expected with CR, many constituents of fatty acid pathways were increased, indicating a shift in metabolic substrate. The majority of features in all pathways associated with fatty acids were increased (Supplementary Table 6). In addition, features in the carnitine shuttle pathway, which is involved in transporting fatty acids across the mitochondrial membrane for β-oxidation, showed an increase with CR at both 20CR and 40CR.

### Distinct Differences at 24AL

**Figure 5.**
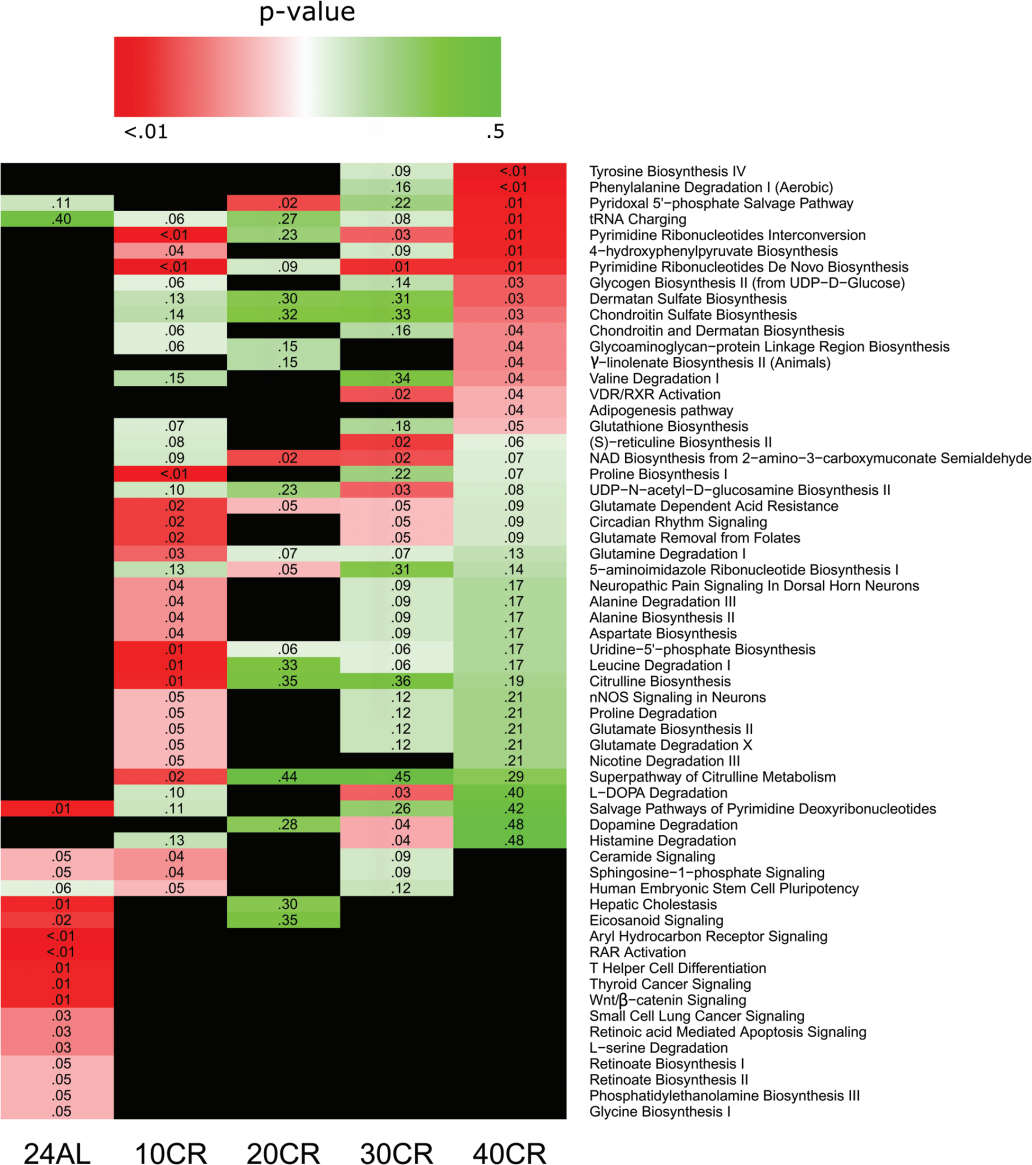
Heat map showing metabolic pathways identified by Ingenuity Pathway Analysis (IPA). Pathways based on metabolites identified by both mummichog and xmsAnnotator. Black indicates pathway not present in group.

Both the IPA and *mummichog* analyses indicated that at a pathway level the 24AL group was distinctly different from the CR groups, whereas between CR groups, there was considerable overlap (Figure 5). These pathways indicated a predisposition to disease and included disease associated pathways such as thyroid cancer and *wnt* signaling. At 24AL relative to 12AL, the vitamin A pathway appeared increased with two significantly increased metabolites and one decreased (*p* = .01). Notably, in contrast to the CR groups, mice under the 24AL control showed significant *decreases* in fatty acid metabolism (Supplementary Table 6).

### Relationship Between Metabolites and Physical Characteristics

We correlated transformed and normalized amino acid intensities with fat-free mass to see if any potential relationships existed between particular amino acids and lean mass (16). We found that plasma amino acids mainly correlated positively with fat-free mass (Supplementary Table 7). However, the amino acid derivative *S*-adenosyl-l-methionine (SAM) significantly negatively correlated with fat-free mass (*R* = −.563, *p* < .001). We also tested for significant correlations between amino acids and hormone levels as well as visceral fat. We saw that significant positive correlations existed between l-leucine and l-glutamate and leptin and insulin. l-Leucine, l-glutamate, and l-methionine also positively correlated with insulin (Supplementary Table 8). In contrast, SAM correlated negatively with insulin (*R* = −.47, *p* = .01) and positively with tumor necrosis factor-α (*R* = −.53, *p* = .01).

We also investigated relationships between plasma aromatic amino acids and cholinergic receptor transcript levels in the hypothalamus, as aromatic amino acids form the precursors of several catecholamines (Supplementary Table 9). We found that tyrosine, the substrate for dopamine synthesis, was negatively correlated with hypothalamic transcriptional levels of *Drd5* (*R* = −.47, *p* = .03), a dopamine receptor expressed in the limbic regions of the brain. l-Phenylalanine and l-tryptophan both significantly negatively correlated with hypothalamic transcriptional levels of *Adrbk2* (Grk3), a beta adrenergic receptor kinase 2 (*R* = .64, *p* = .03 and *R* = .23, *p* = .01, respectively), which is thought to interact with dopamine (26). We also correlated BCAAs, l-leucine and l-valine, with fasting glucose and insulin levels (Supplementary Table 6). We found both BCAAs correlated significantly positively with insulin levels (l-leucine: *R* = .472, *p* = .002; l-valine: *R* = .333, *P* = .036) and fasting glucose correlated significantly positively with l-leucine (*R* = .544, *p* < .001).

We previously found that the stomach, colon, ileum, and cecum were invested in with CR, so we correlated normalized transformed vitamin intensities with the weights of the digestive tract tissues. We found that several metabolites correlated with stomach weight and colon weight (Supplementary Table 10). In addition, vitamin E metabolites correlated negatively with oxidative stress measures including the antioxidant enzyme catalase but positively with hyperperoxides in the blood. Vitamin E metabolites also correlated negatively with leptin (Supplementary Table 11).

### Metabolites and Pathways Linearly Affected by CR

We used the correlation coefficients from correlation of metabolites with level of CR and BH adjusted *p* values for each metabolite to observe pathways that may potentially be associated with life span in the plasma using *mummichog* (Supplementary Table 12) and IPA (Supplementary Table 13). Pathway analysis indicated that positively associated pathways included linoleate, vitamin D3 and E, omega-3, bile acid, and fatty acid metabolism. Negatively associated pathways included the amino acids, glutathione, butanoate and sugar pathways, tyrosine biosynthesis, phenylalanine degradation, S-reticuline biosynthesis, and catecholamine biosynthesis. Metabolites that were significantly associated with the level of CR (Supplementary Table 14) include the sulfonated form of dopamine, dopamine 3-sulfate. This metabolite that is the predominant endogenous form in plasma was significantly decreased at 40CR (logFC = −2.06, adjusted *p* < .007) and correlated negatively with the level of CR (*R* = −.609, adjusted *p* < .001). In addition, the dopamine receptor, *Drd5* measured in the hypothalamus, correlated positively with the level of CR (*R* = .499, *p* = .005). SAM correlated positively with the level of CR (*R* = .483, *p* = .003). Metabolites associated with both arachidonic and linoleic acid pathways were positively correlated with CR, such as anandamide, a key molecule in the endocannabinoid system, which was significantly increased at 20CR, 30CR, and 40CR and correlated positively with CR level (*R* = .513, *p* = .001). In addition, it also correlated positively with Tb (*R* = .480, *p* = .001), and although anandamide did not correlate with total physical activity, it did correlated with food anticipatory activity (*R* = .541, *p* < .001; Supplementary Table 15). In addition, several metabolites that are constituents of bile acid metabolites correlated positively with the level of CR, despite taurine being negatively correlated.

## Discussion

### Graded CR Provides Novel Insights Into the Effects of CR

In contrast to many earlier studies on the effects of CR on life span and health span, which have typically included a single treatment level, here we used graded CR treatment groups. The plasma, as with the liver in our previous study (12), showed an increase in significantly differentially expressed metabolites with increasing CR. However, in contrast to our findings in the liver, we found a large number of plasma metabolites that differed between 12AL and 24AL. This indicates that ad libitum access to food has a greater effect on circulating metabolite levels.

### Changes in Amino Acids With CR May Have an Effect on Catecholamines, Insulin Resistance, Antioxidant Levels, and Longevity

Previous plasma metabolomic analyses on 48-hour 30% CR in C57BL/6 mice reported increases in several amino acids, as well as in creatine, and decreases in choline and glucose (15). Though we did see a significant decrease in glucose at 40CR, our results suggest that the majority of amino acids decreased with increasing CR. This has been seen previously in the serum metabolomic profile of nude mice on 30% restriction for 4 months (27). CR has previously been suggested to increase the breakdown of amino acids, which can be converted to pyruvate for use in gluconeogenesis (28). Although we did not identify pyruvate (molecular weight [MW] = 88) in our screen perhaps due to its small size, we did see a significant decrease in fructose-1,6-bisphosphate, a precursor of glucose at 40CR, which may indicate an increase in gluconeogenesis in organs such as the liver and kidney. We previously reported increased levels of genes linked to gluconeogenesis in the livers of the same mice used in this experiment (29).

Our pathway analyses indicated that there were significant decreases in the tyrosine metabolism pathway and the tryptophan pathway with CR (though tryptophan levels increased). The aromatic amino acids, l-phenylalanine and l-tyrosine, are precursors for dopamine, epinephrine, and norepinephrine, and l-tryptophan is the precursor for serotonin (30). In addition, vitamin B6, the pathway of which was increased at 40CR, acts as a coenzyme for the production of these neurotransmitters (31). We found dopamine 3-sulfate, which is the predominant endogenous form of dopamine in the plasma, negatively associated with the level of CR. In humans, 75% of dopamine sulfate is thought to come from the gastrointestinal tract through sulfoconjugation of dopamine synthesized from l-DOPA, and significantly increases after meals and with foods rich in biogenic amines (32,33).

Dopamine sulfate is thought to function physiologically as a precursor for norepinephrine through conversion to dopamine (32). Although dopamine 3-sulfate did not significantly correlate with the dopamine receptor *Drd5* in the hypothalamus (*R* = −0.48, *p* = .053), l-tyrosine was significantly negatively correlated. *Drd5* itself correlated significantly positively with the level of CR, indicating that as mice became increasingly restricted, the level of this dopamine receptor in the hypothalamus increased. The decrease in substrates and concurrent increase in brain dopamine receptor may indicate that plasma substrates are being converted into dopamine and norepinephrine in the brain. CR has been shown to reduce both the loss of dopaminergic neurons in the substantia nigra of mice (34), and striatal dopamine receptors in the brains of rats (35,36), both of which are associated with reducing age-associated neurodegeneration in conditions such a Parkinson’s disease.

Regarding BCAAs, our plasma results suggest that, l-leucine and l-valine were significantly decreased with increasing CR, indicating that BCAAs may have a negative impact on lifespan in C57BL/6 mice. Previous studies have shown that C57BL/6J mice on a low BCAA diet have improved glucose and pyruvate tolerance and decreased fasting blood glucose and steady insulin levels (37); indeed, the positive relationship that we observed between BCAAs and fasting glucose and insulin levels supports this study.

Weight loss diets have been shown to consistently reduce levels of plasma amino acids in humans including BCAAs (38). It has also been suggested that visceral white adipose tissue may be involved in the accumulation of BCAAs in obese humans and mice (39). We found a strong relationship between several amino acids and visceral fat mass, which mirrors existing studies in obese women that have significantly higher circulating BCAA levels (40). In the latter study, it was also found that glutamate was the strongest predictor of visceral adiposity, and in our study, we also found l-glutamate correlated strongest of the amino acids. A recent study in overweight humans indicated that even mild CR caused a decrease in both plasma l-leucine and l-phenylalanine, in concordance with the findings here (8). The fasting concentrations of BCAAs and aromatic amino acids have been shown to predict future diabetes in healthy, nonglycemic individuals. In some cases, an elevation of these amino acids may occur as much as 12 years before the onset of diabetes (41), which indicates short-term CR may be sufficient to prevent the onset of such metabolic disorders.

### Vitamin E May Be Modulated by Hunger Signaling

Significant changes in vitamin E with CR may occur due to its role as a free-radical scavenging molecule. Also important is its role in membranes, as with age, lower levels of vitamin E have been associated with mitochondrial dysfunction through its protection of membranes from oxidative stress (42). We found that several vitamin E metabolites were positively correlated with reactive oxygen metabolites in the plasma and negatively correlated with catalase in the liver. This may indicate that vitamin E is increasing to mitigate increases in reactive oxygen metabolites in the plasma, as seen previously in rat hepatocytes (43).

Plasma levels of vitamin E bound in circulating lipoproteins can be decreased by insulin infusion in humans, independent of insulin sensitivity, indicating that insulin can affect the oxidant/antioxidant balance through vitamin E (44). In addition, vitamin E tocotrienols, which were increased in our analysis, can improve insulin sensitivity through activation of peroxisome proliferator-activated receptors. The presence of vitamin E stabilizes low-density lipoproteins in the plasma, protecting them from oxidation, which is a symptom of atherosclerosis, and a high intake of vitamin E has been associated with a lower risk of coronary heart disease in women and men (45,46). The breakdown and utilization of adipose tissue for energy during CR, which causes a decrease in both insulin and leptin, may result in a release of bound vitamin and cause an elevation of vitamin E and associated metabolites in the plasma, which then exerts its beneficial effects as an antioxidant and a signaling molecule. In fact, we saw that several vitamin E metabolites correlated significantly negatively with leptin.

### Increases in B Vitamins May Be Improving Age-Associated Declines in Cognition and Increased Oxidative Stress

Vitamin B5 (pantothenic acid) and the reduced form of vitamin B2 (reduced riboflavin) increased with increasing CR and significantly discriminated between feeding groups in the O-PLS-DA. A deficiency of vitamins and minerals has been associated with increased mitochondrial decay and, due to the damaging effects of oxidative stress, aging (47). Deficiencies in vitamin B5, in particular, have been associated with increases in mitochondrial oxidation. Both reduced forms of vitamin B5 and B2 are required for the biosynthesis of heme, which is synthesized in the mitochondria, disruption of which can also contribute to mitochondrial decay and accumulation of iron (48). Specifically, vitamin B2 deficiencies have been seen in elderly human populations, and such insufficiencies have been associated with declining of cognitive function (49,50). This may be due to dietary deficiencies; however, it may also be due to inefficiencies in extracting nutrients from food. The increase in vitamins in the plasma observed here may suggest that nutritional breakdown and absorption is improved in CR animals, which may be associated with investment in the stomach and alimentary tract that we see in the high levels of CR (16). We found that derivatives of vitamin E metabolism and vitamin B2 correlated positively with stomach and colon weight and vitamin B5 correlated positively with stomach weight. However, as well as percent reduction in calories, mice are receiving corresponding reductions in vitamin intake; therefore, increases in vitamin levels could also be characteristic of alterations in gut microbiota. CR has been shown to induce structural changes in gut microbiota in C57BL/6J mice that contribute to vitamin synthesis, particularly B and K vitamins (51,52).

Several changes in C1 and methionine metabolism seen in our results suggest that CR is causing alterations in methionine, SAM, and folate metabolism in a dose-dependent manner. In particular, the increase in SAM and it’s cofactor vitamin B6 and concomitant decrease in precursor methionine, in addition to other constituents of the methionine pathway taurine and glutathione. A previous study has shown that B6 deficiency results in low levels of SAM in the liver. These changes in the plasma, which are particularly apparent at 40CR, further indicate a shift toward production of the methyl-donor SAM. Increases in plasma SAM may be involved in methylation of DNA, proteins, neurotransmitters, and other cellular messengers, coordinating hunger signaling associated changes in the body. In addition, DNA methylation is hugely important for the control of gene expression, whereas hypomethylation of DNA is seen with age and in aging-associated disorders, DNA methylation is thought to be strongly associated with longevity (53).

### Pathways and Metabolites Associated With Lipid β-Oxidation Conserved Between the Plasma and Liver

When we looked at circulating metabolites that had the most important impact on discrimination between the CR treatment groups, both sphinganine-1-phosphate and sphinganine were among these metabolites. Sphinganine significantly increased with increasing CR and sphinganine-1-phosphate was increased in all CR groups. We previously observed that in the liver the sphingolipid/ceramide ratio was a significant part of the hepatic metabolome remodeling with CR (12). Though phospholipid metabolism appears less prominent in the plasma, these metabolites were shown to be increased, as sphingosine-1-phosphate was in the liver, indicating that these metabolites may be secreted into the plasma where they may ultimately be involved in hunger signaling, reducing inflammation and lipid homeostasis. Previous study suggested that in the hypothalamus, S1P regulates energy homeostasis in rats. Microinjection of S1P into the third ventricle of rats increased body temperature and energy expenditure, and microinjection of S1PR1 agonist SEW2871 decreased food intake and activated leptin signaling (54). However, we previously found that when injected peripherally S1P has no effect on body temperature (Tb), food intake, or energy expenditure (12). Furthermore, the fatty acid neurotransmitter and member of the endocannabinoid family anandamide was increased at 20CR, 30CR, and 40CR and has recently been shown to act in the hypothalamus via cannabinoid receptors to control feeding behavior (55). In obese animals, levels of anandamide and other endocannabinoids are increased in both the brain and periphery and administration promotes overeating (56–58). Anandamide may then be part of a suite of peripheral signals produced under restriction that signal the energy balance status to the brain. Signals such as these may in some part explain behaviors such as food anticipatory activity and reduced body temperature in restricted animals (59). Indeed, we saw that anandamide correlated negatively with average Tb over the last 2 weeks of restriction and positively with food anticipatory activity over the same period. However, despite the elevated levels of anandamide, we did not detect any evidence for elevated cannabinoid signaling in the hypothalami of the same mice (25).

Carnitines, increases of which we previously saw in the liver, also increase in the plasma (12), several of which correlated positively with level of CR. Carnitine shuttles long-chain fatty acids across the mitochondrial membrane for β-oxidation and can reduce oxidative stress by preventing the accumulation of toxic long-chain fatty acyl-CoA metabolites (60). Pathway analysis indicated carnitine shuttle components were increased with CR; however, in contrast to the liver, l-carnitine appeared significantly decreased at 40CR. As l-carnitine shuttles long-chain fatty acids across the mitochondrial membrane, and mammalian red blood cells do not contain mitochondria, l-carnitine may be moving from the plasma into the liver and muscle for use in fatty acid oxidation. We found that the levels of O-acetylcarnitine, which increased with increasing CR, correlated positively with levels of O-acetylcarnitine in the liver. As carnitines have been implicated in insulin sensitivity and inflammation, it may be that carnitine has a distinct role in the plasma compared with the liver (61).

Although taurine, a constituent of bile, was reduced with increasing CR, circulating levels of 7α-hydroxycholesterol, 5β-cholestane-3α,7α-diol, 5β-cholestane-3α,7α,26-triol, 5β-cyprinolsulfate, and 7α-hydroxy-3-oxo-4-cholestenoate, which are involved in primary bile acid synthesis, showed a graded increase with increasing CR and were all decreased at 24AL. This may indicate that the plasma, although not involved in lipid breakdown, transports the constituents of bile acids to organs such as the liver, whereas products, such as taurine, are secreted into the plasma for disposal. In the gut, bile acids break down large fat globules into smaller droplets and promote their absorption through formation of micelles. This helps stimulate energy metabolism and improves insulin sensitivity (62). CR has been shown to reverse the decline in the bile acid synthesis pathway with age in female C57BL/6 mice (63). There is also increasing evidence that bile acids play a role as signaling molecules through G-protein-coupled receptors in the liver and may result in amelioration of obesity and diabetes in mice (64).

We saw a decrease in glucose at 30CR and 40CR and a graded increase in the fatty acid-CoA molecules, 3-hydroxyoctadecanoyl-CoA and 2-hydroxyphytanoyl-CoA, which indicates a shift toward the fatty acid entry to the citric acid cycle and away from glycolysis. As with l-carnitine, these molecules may be in transit to the liver or muscles for breakdown. This result is consistent with the theory that CR results in metabolic reprogramming involving a move away from glycolysis and toward fatty acid β-oxidation.

However, we saw no significant changes in metabolites involved in both the urea cycle and production of ketone bodies, two processes commonly associated with breakdown of amino acids and fatty acid oxidation during acute starvation (65,66). There may be several explanations for this. First, some of the products of amino acid metabolism and ketogenesis were too small to be detected. For example, urea (MW = 60.06) and acetone (MW = 58.09) were both below the approximate MW detection limit of 85 for the liquid chromatography–mass spectrometry we used. However, other products of these pathways were large enough to be detected. We did detect l-arginine (MW = 174.2) and l-citrulline (MW = 175.2) but neither were correlated with the levels of restriction. We did not detect the main ketones, acetoacetate and β-hyroxybutyrate, despite the sizes of these compounds being detectable (MW 102 and 104, respectively). This suggests that ketogenesis was minimal. Although this seems to contrast with several other studies, the previous study involved mice undergoing much shorter periods of CR (15,66) relative to the 3 months in the present study. This is important as the mice in our study showed two distinct stages of metabolic response: a dynamic phase when both fat and lean tissues were withdrawn, which lasted about 30 days, followed by a period of stability when body weight and composition were constant (16). During this period, the mice were back in energy balance between their energy demands and their ingested food, which was predominantly carbohydrate. Because the mice were sampled after 60 days in this stable phase, we would not expect significant utilization of amino acids or triglycerides from the tissues to still be occurring. Consequently, we would also not anticipate significant levels of the byproducts of amino acid degradation, and ketone bodies in the plasma. This was also consistent with the absence of significant changes in such compounds in the liver metabolome of the same mice (12). Although there does appear evidence of significant upregulation of β-oxidation of fatty acids in these mice (29,67), this was also matched by upregulation of the hepatic tricarboxylic acid (TCA) cycle activity, and this may explain why ketones were not generated in significant amounts from fatty acid oxidation. When individuals reach steady state, it is likely there is a cycle in metabolite usage through the 24-hour day. This would require a dependence on carbohydrates via glycolysis directly from the food during the night, combined with significant lipogenesis to convert ingested CHO to stored fat (68), which can then be utilized via β-oxidation during the day, probably supplemented by gluconeogenesis, once the food has run out, which was the time point at which we sacrificed them. Finally, metabolite detection between the serum and plasma differs significantly in humans and rats, in particular concentrations of metabolites tend to be smaller in the plasma than in the serum, which has been attributed to the volume displacement effect. In particular, ornithine (urea cycle), arginine (urea cycle), arachidonic acid (phospholipid), and β-hydroxybutyrate (ketone body) are lower in the plasma than in the serum (69,70).

### Distinct Differences Exist at 24AL and at Differing Levels of CR

As can be seen in Figure 5, metabolic pathway changes were not consistent across treatment groups, and although some metabolites and pathways have a linear relationship with CR, others do not. A particular absence of significant pathways appears at 20CR, which may indicate a threshold level for metabolic effects, prior to a range of significant changes at 30CR and 40CR. This is consistent with previous metabolic analyses in the liver, which indicated that the relationship between CR and metabolite level is not always linear (6). What is particularly of note is the large number of significantly altered pathways at both 10CR and 40CR, of which there are very few overlaps, which may indicate a stable state shift at the extremities, with greater flux in pathway changes between 20CR and 30CR.

Pathway analyses indicated that many pathways significantly altered in 24AL are unmistakeably different from those of the CR groups (Figure 5). Several of these pathways were associated with cancer, including thyroid and lung cancer cell signaling and wnt/β cantenin signaling, this suggests metabolites that are being altered in the 24AL group may be involved in tumorigenesis. Many of the other significant pathways involved fatty acid metabolism, including glycerophospholipid, linoleate, and omega-3 fatty acid metabolism and fatty acid activation were altered. In addition to this, we saw changes in vitamin B9, vitamin A, vitamin E metabolism as well as sialic acid and selanoamino metabolism.

It is important to note that changes in metabolic pathways may also depend on the strain of mouse, as the longevity impact of CR and changes in the metabolome are known to be related to genetic background (6,71). In addition, although we only used male mice in this study, sex differences in the metabolic profiles of male and female mice have been observed, in particular, in groups of metabolites such as the BCAAs (6,72).

## Conclusion

Circulating levels of sphingolipids, carnitines, and bile acids were all significantly altered in the plasma with CR, many showing a graded effect, reflecting similar changes observed previously in the livers of the same mice (12). Changes in the components of these pathways with CR, however, appeared less significant than in the liver, possibly due to the primary transport role of plasma. As seen previously, many of the metabolites were altered in a graded fashion, highlighting their potential relevance to longevity. In contrast to the smaller effects in plasma of sphingolipids, carnitines, and bile acids, the amino acids showed greater changes in the plasma than in the liver. BCAAs were all decreased in the plasma, consistent with previous suggestions that reduced BCAAs reduce insulin resistance and fasting glucose. We also saw notable changes in the circulating levels of vitamins, with nearly every major group being elevated at higher levels of CR despite their dietary supply being reduced. This included several elements of the vitamin E complex and most B vitamins. This result may be due to an increasing efficiency of the gut through investment in size and changes in gut microbiota to breakdown and absorb key nutrients from their limited food intake. Increased vitamin E is associated with improved mitochondrial and membrane function, which supports the suggestion that CR may improve metabolism through promoting utilization of lipids during CR and regulating energy homeostasis.

## Funding

The study was supported by the UK Biotechnology and Biological Sciences Research Council BBSRC (BB/G009953/1 and BB/J020028/1 to J.R.S.) and a studentship of C.L.G. from the BBSRC EastBio Doctoral Training Partnership. C.L.G. received support from the laboratory of D.P.; D.P. was supported in part by NIH grant AGO49494.

## Data Accessibility

Work toward having all the data from this series of papers online is currently ongoing. All significant metabolites in relation to CR manipulation are listed in Supplementary Material S1. Data on the nonsignificant metabolites are freely available for anyone who requests it from the corresponding author at j.speakman@abdn.ac.uk.

## Conflict of Interest

None reported.

## Supplementary Material

Supplementary Material
