# Supplementary material for: The Effects of Graded Levels of Calorie Restriction: XIII. Global Metabolomics Screen Reveals Graded Changes in Circulating Amino Acids, Vitamins, and Bile Acids in the Plasma of C57BL/6 Mice
Source: J Gerontol A Biol Sci Med Sci. 2018 Apr 30;74(1):16–26. doi: 10.1093/gerona/gly058 (PMC6298180; doi:10.1093/gerona/gly058)
Supplement: Supplementary Material [file gly058_suppl_supplementary_materials.docx]

Supplementary material

S1: Additional information on experimental design

The 12AL group was used as the control to account for the potential for the ‘time since last meal’ effect caused the confounding effects of food consumption immediate prior to culling which may occur in the 24AL group (Speakman & Mitchell 2011; Sohal & Forster 2014). We initiated CR at 20 weeks, which is approximately equivalent to early adulthood in humans. This was done to avoid any effect of CR on development whilst retaining effectiveness of increasing lifespan (Yu et al. 1985). Mice were fed CR (or AL) diets for three months, before being sacrificed at eight months of age. Body mass and food intake were recorded daily, immediately prior to feeding. On day of cull food was removed from both the AL groups and mice were culled approximately 7 hours prior to lights out between 14:00 to 18:00 by a terminal CO_2_ overdose. Blood sample was collected by heart puncture, spun and aliquoted into heparin tubes and stored at -80 °C.

S2: Additional information on dual chromatography – Fourier transform mass spectrometry (DC-FTMS)

At the beginning of each sample set, standards from the National Institute of Standards and Technology were run. At the beginning, end and every 20 samples a quality control of pooled plasma was run. Every sample was spiked with an internal standard mixture that consists of 5 stable isotopes of known concentrations. To enhance metabolite detection we used dual chromatography, utilising both anion exchange (AE) and reverse phase (C18) columns to separate samples. A switching valve allowed data to be collected alternately between the AE and C18 column. Data was converted using Xcalibur file converter software (Thermo Fischer, San Diego, CA) and peak extraction and ion intensities was analysed using apLCMS in R. This provided m/z, RT and ion intensity values.

S3: Additional information on O-PLS-DA analysis and validation steps

O-PLS-DA allows us to discriminate between treatment groups in multivariate data and to determine the most influential metabolites based on loadings, which function as good ‘summaries’ of the variables' influence on the model (Westerhuis et al. 2010; Worley & Powers 2013). We compared our model fit to random chance by the generation of 1000 permuted models, and splitting the data into a pseudo training/test set to simulate root mean square error of prediction (RMSEP). Test/training was randomly assigned to the 46 samples in a 12:34 ratio, 1000 times. We compared their performance statistics to our own.

S4: Additional information regarding pathway analyses.

IPA allowed us to take advantage of the Ingenuity® Knowledge Base, a repository of biological and chemical information from the literature. The Ingenuity® Knowledge Base also contains information about the cause-effect relationships between molecules, diseases and biological functions. Unlike IPA, for which a metabolite ID is needed to access information, *mummichog* is able to predict functional activity from mass to charge (m/z) values without *a priori* identification of metabolites.

Supplementary Tables

## Table 1: Number of significantly differentially expressed (SDE) metabolites, relative to 12AL control for each CR treatment level and the 24AL group in the AE column (Benjamini Hochberg adjusted P ≤ 0.02).

|  | 24AL | 10CR | 20CR | 30CR | 40CR |
| --- | --- | --- | --- | --- | --- |
| Up | 144 | 82 | 176 | 215 | 264 |
| Unchanged | 1496 | 1573 | 1416 | 1362 | 1275 |
| Down | 62 | 47 | 110 | 125 | 163 |

## **Table 2: Number of significantly differentially expressed (SDE) metabolites, relative to 12AL control for each CR treatment level and the 24AL group in the C18 column (P ≤ 0.05).**

|  | 24AL | 10CR | 20CR | 30CR | 40CR |
| --- | --- | --- | --- | --- | --- |
| Up | 57 | 28 | 150 | 125 | 163 |
| Unchanged | 1156 | 1200 | 1030 | 1049 | 982 |
| Down | 39 | 24 | 72 | 78 | 107 |

**Table 3: Significant identified metabolites that significantly discriminate between treatment groups based on O-PLS-DA model.**

| Name | Model weight | Pcorr | Loadings | P-value | |  |
| --- | --- | --- | --- | --- | --- | --- |
| 13(S)-HPOT | 0.050 | 0.889 | 0.050 | | <0.001 | |
| Reduced riboflavin | 0.046 | 0.842 | 0.046 | | <0.001 | |
| gama-tocotrienol | 0.045 | 0.792 | 0.045 | | <0.001 | |
| Deacetylisoipecoside | 0.045 | 0.799 | 0.045 | | <0.001 | |
| N-(12S-hydroxy-5Z,8Z,10E,14Z-eicosatetraenoyl)-glycine | 0.045 | 0.780 | 0.045 | | <0.001 | |
| 9'-carboxy-gama-chromanol | 0.045 | 0.772 | 0.045 | | <0.001 | |
| 6-trans-leukotriene B4 | 0.044 | 0.803 | 0.044 | | <0.001 | |
| 11'-carboxy-gama-tocotrienol | 0.044 | 0.767 | 0.044 | | <0.001 | |
| Karakoline | 0.043 | 0.776 | 0.043 | | <0.001 | |
| 4alpha-Methylzymosterol-4-carboxylate | 0.043 | 0.708 | 0.043 | | <0.001 | |
| Tomentolide A | 0.043 | 0.746 | 0.043 | | <0.001 | |
| Anandamide | 0.042 | 0.731 | 0.042 | | <0.001 | |
| beta-D-Glucosyl crocetin | 0.042 | 0.754 | 0.042 | | <0.001 | |
| 13'-hydroxy-gama-tocotrienol | 0.041 | 0.768 | 0.041 | | <0.001 | |
| Protoporphyrinogen IX | 0.041 | 0.717 | 0.041 | | <0.001 | |
| Euchrenone a6 | 0.041 | 0.752 | 0.041 | | <0.001 | |
| Phytosphingosine | 0.041 | 0.744 | 0.041 | | <0.001 | |
| Glucosylgalactosyl hydroxylysine | 0.041 | 0.671 | 0.041 | | <0.001 | |
| Docosanoic acid | 0.041 | 0.755 | 0.041 | | <0.001 | |
| 12(S)-hydroxyarachidonylethanolamide | 0.040 | 0.752 | 0.040 | | <0.001 | |
| Myxalamid D | 0.040 | 0.751 | 0.040 | | <0.001 | |
| 3alpha,7alpha,26-Trihydroxy-5beta-cholestane | 0.040 | 0.755 | 0.040 | | <0.001 | |
| Sphinganine | 0.040 | 0.608 | 0.040 | | <0.001 | |
| 7R-hydroxy-hexadecanoic acid | 0.040 | 0.632 | 0.040 | | <0.001 | |
| LysoPC(15:0) | 0.039 | 0.707 | 0.039 | | <0.001 | |
| Pantothenate | 0.039 | 0.699 | 0.039 | | <0.001 | |
| 3beta-Hydroxy-4beta-methyl-5alpha-cholest-7-ene-4alpha-carboxylate | 0.039 | 0.643 | 0.039 | | <0.001 | |
| Linoleate | 0.038 | 0.597 | 0.038 | | <0.001 | |
| 7alpha-Hydroxycholesterol | 0.038 | 0.597 | 0.038 | | <0.001 | |
| Glaucarubin | 0.038 | 0.664 | 0.038 | | <0.001 | |
| (-)-Salvisyriacolide | 0.038 | 0.631 | 0.038 | | <0.001 | |
| 3alpha,7alpha,12alpha,26,27-pentahydroxy-5beta-cholestane 26-sulfate | 0.038 | 0.686 | 0.038 | | <0.001 | |
| nisinate | 0.037 | 0.666 | 0.037 | | <0.001 | |
| heptadecanoyl carnitine | 0.037 | 0.639 | 0.037 | | <0.001 | |
| 13-cis-retinoate | 0.037 | 0.732 | 0.037 | | <0.001 | |
| 13'-hydroxy-alpha-tocotrienol | 0.037 | 0.677 | 0.037 | | <0.001 | |
| N-(9Z-octadecenoyl)-taurine | 0.036 | 0.758 | 0.036 | | <0.001 | |
| (7E)-(1R,3R,20S)-2-methylene-19,nor-20-dihomo-9,10-seco-5,7-pregnadien-1,3-diol | 0.036 | 0.576 | 0.036 | | <0.001 | |
| 13-OxoODE | 0.036 | 0.701 | 0.036 | | <0.001 | |
| 11-chloro-12-hydroxy-octadecanoic acid | 0.036 | 0.664 | 0.036 | | <0.001 | |
| Aurasperone D | 0.036 | 0.614 | 0.036 | | <0.001 | |
| Pyridoxamine phosphate | 0.036 | 0.524 | 0.036 | | 0.002 | |
| alpha-Tocotrienol | 0.035 | 0.584 | 0.035 | | <0.001 | |
| methyl 5S,6S,12R-triacetoxy-9-oxo-10-chloro-7Z,10E,14Z,17Z-prostatetraenoate-cyclo[8,12R] | 0.035 | 0.564 | 0.035 | | 0.001 | |
| 15-hydroxynorandrostene-3,17-dione glucuronide | 0.035 | 0.587 | 0.035 | | <0.001 | |
| tetradecanoyl carnitine | 0.035 | 0.535 | 0.035 | | 0.001 | |
| Luteolin 7,3'-dimethyl ether 4'-glucoside | 0.034 | 0.524 | 0.034 | | 0.002 | |
| 3-Hydroxyoctadecanoyl-CoA | 0.034 | 0.551 | 0.034 | | 0.001 | |
| FADH2 | 0.034 | 0.448 | 0.034 | | 0.009 | |
| O-(4-Hydroxy-3,5-diidophenyl)-3,5-diiodo-L-tyrosine | 0.034 | 0.516 | 0.034 | | 0.002 | |
| Pentosidine | 0.034 | 0.624 | 0.034 | | <0.001 | |
| 3alpha,7alpha-Dihydroxy-5beta-cholestane | 0.034 | 0.582 | 0.034 | | <0.001 | |
| Calcidiol | 0.034 | 0.520 | 0.034 | | 0.002 | |
| 9'-carboxy-alpha-chromanol | 0.033 | 0.716 | 0.033 | | <0.001 | |
| 2-Hydroxyphytanoyl-CoA | 0.033 | 0.627 | 0.033 | | <0.001 | |
| Calcitriol | 0.033 | 0.643 | 0.033 | | <0.001 | |
| N-(12S-hydroxy-5Z,8Z,10E,14Z-eicosatetraenoyl)-alanine | 0.033 | 0.683 | 0.033 | | <0.001 | |
| Succinylcholine | 0.033 | 0.524 | 0.033 | | 0.002 | |
| 13-Deoxydaunorubicin | 0.033 | 0.573 | 0.033 | | <0.001 | |
| Methotrimeprazine | 0.033 | 0.527 | 0.033 | | 0.001 | |
| 3alpha-Hydroxy-5beta-pregnane-20-one | 0.033 | 0.558 | 0.033 | | 0.001 | |
| 5,2'-Dihydroxy-6,7,8,6'-tetramethoxyflavanone | 0.032 | 0.606 | 0.032 | | <0.001 | |
| Geranylgeranyl diphosphate | 0.032 | 0.453 | 0.032 | | 0.008 | |
| (6Z,9Z,12Z)-Octadecatrienoic acid | 0.032 | 0.592 | 0.032 | | <0.001 | |
| 10,11-dihydro-12R-hydroxy-leukotriene E4 | 0.032 | 0.549 | 0.032 | | 0.001 | |
| 13(S)-HODE | 0.031 | 0.555 | 0.031 | | 0.001 | |
| Benzyl viologen | 0.031 | 0.572 | 0.031 | | <0.001 | |
| (5Z,7E)-25-hydroxy-9,10-seco-5,7,10(19)-cholestatrien 3R-(1,2-epoxy-propyl)ether | 0.031 | 0.563 | 0.031 | | 0.001 | |
| Ergovaline | 0.031 | 0.494 | 0.031 | | 0.003 | |
| 9S,10S-dihydroxyoctadecanoic acid | 0.029 | 0.395 | 0.029 | | 0.027 | |
| 15-oxo-hexadecanoic acid | 0.029 | 0.511 | 0.029 | | 0.002 | |
| O-Acetylcarnitine | 0.029 | 0.497 | 0.029 | | 0.003 | |
| (9Z,11E)-(13S)-13-Hydroperoxyoctadeca-9,11-dienoic acid | 0.029 | 0.541 | 0.029 | | 0.001 | |
| L-Acetylcarnitine | 0.029 | 0.492 | 0.029 | | 0.003 | |
| N-octadecanoyl-tyrosine | 0.028 | 0.550 | 0.028 | | 0.001 | |
| Diacetylnivalenol | 0.028 | 0.531 | 0.028 | | 0.001 | |
| Prodelphinidin B6 | 0.028 | 0.510 | 0.028 | | 0.002 | |
| Diafenthiuron | 0.028 | 0.407 | 0.028 | | 0.021 | |
| Geranioloxyalatum flavone | 0.028 | 0.531 | 0.028 | | 0.001 | |
| Oplophorus luciferin | 0.027 | 0.460 | 0.027 | | 0.007 | |
| 7alpha-Hydroxy-3-oxo-4-cholestenoate | 0.027 | 0.487 | 0.027 | | 0.004 | |
| 9-[1'E,3'Z,6'Z-trien-1'-yloxy]-non-8E-enoic acid | 0.026 | 0.364 | 0.026 | | 0.045 | |
| 5-Dehydroepisterol | 0.025 | 0.386 | 0.025 | | 0.031 | |
| Vitamin K | 0.025 | 0.577 | 0.025 | | <0.001 | |
| (5Z,7E)-(3S)-3-azido-9,10-seco-5,7,10(19)-cholestatrien-25-ol | 0.025 | 0.439 | 0.025 | | 0.011 | |
| N6,N6,N6-Trimethyl-L-lysine | 0.025 | 0.453 | 0.025 | | 0.008 | |
| (+)-gama-tocopherol | 0.025 | 0.433 | 0.025 | | 0.013 | |
| GW1843 | 0.024 | 0.402 | 0.024 | | 0.024 | |
| S-Adenosyl-L-methionine | 0.023 | 0.424 | 0.023 | | 0.015 | |
| Butralin | 0.023 | 0.488 | 0.023 | | 0.004 | |
| Bromobenzene | 0.023 | 0.394 | 0.023 | | 0.028 | |
| 4-oxo-9-cis-retinoate | 0.023 | 0.359 | 0.023 | | 0.048 | |
| 6-(Dibromomethylene)-17beta-hydroxy-androst-4-en-3-one propionate | 0.023 | 0.412 | 0.023 | | 0.019 | |
| (5Z,7E)-(1S,3R)-24-methyl-9,10-seco-5,7,10(19),22-ergostatetraene-1,3-diol | 0.022 | 0.367 | 0.022 | | 0.043 | |
| Platensimycin | 0.021 | 0.373 | 0.021 | | 0.039 | |
| Nomega,Nomega'-Dimethyl-L-arginine | 0.021 | 0.516 | 0.021 | | 0.002 | |
| Fusarenon-X | 0.020 | 0.400 | 0.020 | | 0.025 | |
| Robustic acid methyl ether | 0.020 | 0.363 | 0.020 | | 0.046 | |
| gamma-Linolenoyl-CoA | 0.019 | 0.533 | 0.019 | | 0.001 | |
| 18-carboxy-dinor-LTE4 | 0.019 | 0.376 | 0.019 | | 0.037 | |
| 7'-carboxy-alpha-chromanol | 0.019 | 0.468 | 0.019 | | 0.006 | |
| SN-38 | 0.016 | 0.358 | 0.016 | | 0.049 | |
| Fructoselysine 6-phosphate | 0.015 | 0.359 | 0.015 | | 0.048 | |
| 3alpha-Hydroxy-5beta-cholanate | -0.006 | -0.366 | -0.006 | | 0.043 | |
| Methaphenilene | -0.013 | -0.475 | -0.013 | | 0.005 | |
| alpha-linolenyl carnitine | -0.014 | -0.417 | -0.014 | | 0.018 | |
| 20-CoA-20-oxo-18R-hydroxyleucotriene B4 | -0.015 | -0.361 | -0.015 | | 0.047 | |
| Sulfolithocholylglycine | -0.016 | -0.400 | -0.016 | | 0.025 | |
| S-(4-Bromophenyl)-L-cysteine | -0.016 | -0.379 | -0.016 | | 0.035 | |
| Methylarsonate | -0.017 | -0.482 | -0.017 | | 0.004 | |
| L-Proline | -0.017 | -0.382 | -0.017 | | 0.033 | |
| 6-Phospho-2-dehydro-D-gluconate | -0.017 | -0.447 | -0.017 | | 0.009 | |
| Glucobrassicin | -0.017 | -0.357 | -0.017 | | 0.049 | |
| Tetrahydrobiopterin | -0.017 | -0.362 | -0.017 | | 0.046 | |
| Cefprozil | -0.018 | -0.395 | -0.018 | | 0.027 | |
| L-Iduronate 2-sulfate | -0.018 | -0.369 | -0.018 | | 0.041 | |
| Glucoconringiin | -0.018 | -0.368 | -0.018 | | 0.042 | |
| Delphinine | -0.018 | -0.398 | -0.018 | | 0.025 | |
| kinetensin | -0.019 | -0.357 | -0.019 | | 0.049 | |
| (8Z,11Z,14Z)-Icosatrienoic acid | -0.020 | -0.472 | -0.020 | | 0.005 | |
| Palmitelaidic acid | -0.020 | -0.363 | -0.020 | | 0.045 | |
| (3S)-3-hydroxydodec-cis-6-enoyl-CoA | -0.020 | -0.361 | -0.020 | | 0.047 | |
| 1,2-Dihydronaphthalene-1,2-diol | -0.020 | -0.371 | -0.020 | | 0.040 | |
| Deoxyuridine | -0.020 | -0.434 | -0.020 | | 0.012 | |
| 1-(5'-Phosphoribosyl)-5-formamido-4-imidazolecarboxamide | -0.020 | -0.384 | -0.020 | | 0.032 | |
| Kanzakiflavone 1 | -0.020 | -0.375 | -0.020 | | 0.037 | |
| I-Urobilinogen | -0.020 | -0.364 | -0.020 | | 0.044 | |
| Glycyrol | -0.021 | -0.388 | -0.021 | | 0.030 | |
| D-Ribose 1-diphosphate | -0.021 | -0.358 | -0.021 | | 0.049 | |
| Thiopurine | -0.021 | -0.358 | -0.021 | | 0.049 | |
| Sulfocarbathione | -0.022 | -0.386 | -0.022 | | 0.031 | |
| Sucrose | -0.022 | -0.436 | -0.022 | | 0.012 | |
| L-Cystine | -0.022 | -0.374 | -0.022 | | 0.038 | |
| Carfentrazone-ethyl | -0.022 | -0.432 | -0.022 | | 0.013 | |
| Quercetin 3,7-diglucuronide | -0.022 | -0.407 | -0.022 | | 0.022 | |
| (5Z,7E)-(1R,3R)-1,25-difluoro-9,10-seco-5,7,10(19)-cholestatriene-3-ol | -0.022 | -0.500 | -0.022 | | 0.003 | |
| Melibiitol | -0.022 | -0.443 | -0.022 | | 0.010 | |
| Dichlorvos | -0.023 | -0.465 | -0.023 | | 0.006 | |
| Tetrahydropapaveroline | -0.023 | -0.523 | -0.023 | | 0.002 | |
| Propinol adenylate | -0.023 | -0.448 | -0.023 | | 0.009 | |
| Pyridoxine phosphate | -0.023 | -0.406 | -0.023 | | 0.022 | |
| S-Succinyldihydrolipoamide | -0.023 | -0.448 | -0.023 | | 0.009 | |
| Famphur | -0.023 | -0.368 | -0.023 | | 0.042 | |
| Phenolic phosphate | -0.023 | -0.477 | -0.023 | | 0.005 | |
| 3-Hydroxydodecanoic acid | -0.023 | -0.445 | -0.023 | | 0.010 | |
| Rifamycin | -0.023 | -0.358 | -0.023 | | 0.049 | |
| S-[2-(N7-Guanyl)ethyl]-N-acetyl-L-cysteine | -0.023 | -0.432 | -0.023 | | 0.013 | |
| UDP-D-xylose | -0.024 | -0.409 | -0.024 | | 0.021 | |
| Felbamate | -0.024 | -0.362 | -0.024 | | 0.046 | |
| (2S)-5,7,2'-Trihydroxy-8-(5-hydroxy-2-isopropenyl-5-methylhexyl)flavanone | -0.024 | -0.380 | -0.024 | | 0.034 | |
| N3'-Acetylapramycin | -0.024 | -0.430 | -0.024 | | 0.013 | |
| Cefaloglycin | -0.025 | -0.391 | -0.025 | | 0.029 | |
| Selenocystathionine | -0.025 | -0.459 | -0.025 | | 0.007 | |
| 5-Amino-6-(5'-phospho-D-ribitylamino)uracil | -0.025 | -0.475 | -0.025 | | 0.005 | |
| 4,6,3',4'-Tetramethoxyaurone | -0.025 | -0.492 | -0.025 | | 0.003 | |
| 2-Methylhippuric acid | -0.025 | -0.427 | -0.025 | | 0.014 | |
| Chlorfluazuron | -0.025 | -0.452 | -0.025 | | 0.009 | |
| 10-Deacetyl-2-debenzoylbaccatin III | -0.026 | -0.557 | -0.026 | | 0.001 | |
| Ikarisoside B | -0.026 | -0.459 | -0.026 | | 0.007 | |
| 3,5-Diiodo-L-tyrosine | -0.026 | -0.390 | -0.026 | | 0.029 | |
| dITP | -0.027 | -0.577 | -0.027 | | <0.001 | |
| Ipazine | -0.027 | -0.465 | -0.027 | | 0.006 | |
| Glutathione | -0.027 | -0.496 | -0.027 | | 0.003 | |
| UDP | -0.027 | -0.391 | -0.027 | | 0.029 | |
| GTP | -0.027 | -0.513 | -0.027 | | 0.002 | |
| L-alpha-Acetyl-N,N-dinormethadol | -0.027 | -0.496 | -0.027 | | 0.003 | |
| Peptide 2-(3-carboxy-3-aminopropyl)-L-histidine | -0.027 | -0.464 | -0.027 | | 0.007 | |
| stearidonyl carnitine | -0.027 | -0.435 | -0.027 | | 0.012 | |
| 3-O-alpha-Mycarosylerythronolide B | -0.028 | -0.495 | -0.028 | | 0.003 | |
| 1-Diphosinositol pentakisphosphate | -0.028 | -0.381 | -0.028 | | 0.034 | |
| Enterolactone | -0.028 | -0.569 | -0.028 | | <0.001 | |
| Uridine | -0.029 | -0.465 | -0.029 | | 0.006 | |
| Creatine | -0.029 | -0.426 | -0.029 | | 0.015 | |
| L-Glutamyl 5-phosphate | -0.029 | -0.525 | -0.029 | | 0.002 | |
| Prothiofos | -0.029 | -0.562 | -0.029 | | 0.001 | |
| Thebaine | -0.029 | -0.479 | -0.029 | | 0.005 | |
| Archangelolide | -0.029 | -0.526 | -0.029 | | 0.002 | |
| D-Fructose 1,6-bisphosphate | -0.030 | -0.556 | -0.030 | | 0.001 | |
| dADP | -0.030 | -0.504 | -0.030 | | 0.003 | |
| Chondroitin | -0.030 | -0.511 | -0.030 | | 0.002 | |
| 1,2-didecanoyl-sn-glycero-3-phospho-(1'-sn-glycerol) | -0.030 | -0.416 | -0.030 | | 0.018 | |
| UTP | -0.030 | -0.629 | -0.030 | | <0.001 | |
| Orotidine 5'-phosphate | -0.031 | -0.540 | -0.031 | | 0.001 | |
| Estrone | -0.031 | -0.619 | -0.031 | | <0.001 | |
| N-Acetylmuramic acid 6-phosphate | -0.031 | -0.571 | -0.031 | | <0.001 | |
| Cocaine | -0.031 | -0.659 | -0.031 | | <0.001 | |
| (S)-Hydroxydecanoyl-CoA | -0.031 | -0.552 | -0.031 | | 0.001 | |
| Dihydropicromycin | -0.031 | -0.643 | -0.031 | | <0.001 | |
| Estriol 3-sulfate 16-glucuronide | -0.031 | -0.546 | -0.031 | | 0.001 | |
| L-Tyrosine | -0.032 | -0.660 | -0.032 | | <0.001 | |
| dTDP-L-oleandrose | -0.032 | -0.559 | -0.032 | | 0.001 | |
| 6-Hydroxycyanidin 3-rutinoside | -0.032 | -0.633 | -0.032 | | <0.001 | |
| L-Valine | -0.032 | -0.528 | -0.032 | | 0.001 | |
| Aminoimidazole ribotide | -0.032 | -0.659 | -0.032 | | <0.001 | |
| (R)-5-Diphosphomevalonate | -0.032 | -0.616 | -0.032 | | <0.001 | |
| Azamethiphos | -0.033 | -0.536 | -0.033 | | 0.001 | |
| Neosaxitoxin | -0.033 | -0.620 | -0.033 | | <0.001 | |
| Cinnavalininate | -0.033 | -0.639 | -0.033 | | <0.001 | |
| L-Phenylalanine | -0.034 | -0.622 | -0.034 | | <0.001 | |
| L-Methionine | -0.034 | -0.440 | -0.034 | | 0.011 | |
| 1-(1Z-hexadecenyl)-2-(6Z,9Z,12Z,15Z-octadecatetraenoyl)-glycero-3-phospho-(1'-myo-inositol) | -0.034 | -0.572 | -0.034 | | <0.001 | |
| Pikromycin | -0.034 | -0.446 | -0.034 | | 0.010 | |
| 2-Amino-3-carboxymuconate semialdehyde | -0.034 | -0.626 | -0.034 | | <0.001 | |
| (5Z,13E)-11alpha-Hydroxy-9,15-dioxoprost-13-enoate | -0.034 | -0.543 | -0.034 | | 0.001 | |
| 2'''-N-Acetyl-6'''-deamino-6'''-hydroxyneomycin C | -0.035 | -0.620 | -0.035 | | <0.001 | |
| Glutathionylspermine | -0.035 | -0.580 | -0.035 | | <0.001 | |
| 5-Methylcytidine | -0.036 | -0.706 | -0.036 | | <0.001 | |
| 3-Hydroxy-4-methoxy-8,9-methylenedioxycoumestan | -0.036 | -0.609 | -0.036 | | <0.001 | |
| 3,4-Dihydro-3-hydroxy-4-S-glutathionyl bromobenzene | -0.036 | -0.638 | -0.036 | | <0.001 | |
| Cholest-4-en-3-one | -0.036 | -0.452 | -0.036 | | 0.009 | |
| Melosatin B | -0.036 | -0.699 | -0.036 | | <0.001 | |
| Dopamine 3-O-sulfate | -0.036 | -0.684 | -0.036 | | <0.001 | |
| 4a-Hydroxytetrahydrobiopterin | -0.036 | -0.593 | -0.036 | | <0.001 | |
| LysoPC(22:6(4Z,7Z,10Z,13Z,16Z,19Z)) | -0.037 | -0.720 | -0.037 | | <0.001 | |
| Amisulbrom | -0.037 | -0.678 | -0.037 | | <0.001 | |
| L-Carnitine | -0.037 | -0.653 | -0.037 | | <0.001 | |
| CDP-ethanolamine | -0.038 | -0.763 | -0.038 | | <0.001 | |
| Graveoline | -0.039 | -0.590 | -0.039 | | <0.001 | |
| Costatol | -0.039 | -0.686 | -0.039 | | <0.001 | |
| Ammonium tetrathiocyanato-diamminechromate(III) | -0.039 | -0.660 | -0.039 | | <0.001 | |
| neuromedin N (1-4) | -0.041 | -0.509 | -0.041 | | 0.002 | |
| L-Glutamate | -0.042 | -0.754 | -0.042 | | <0.001 | |
| Mycinamicin VII | -0.044 | -0.482 | -0.044 | | 0.004 | |
| L-Leucine | -0.044 | -0.797 | -0.044 | | <0.001 | |

## **Table 4: Mummichog pathway enrichment for combined AE and C18 columns. Fold-changes and P-values relative to the 12AL control group entered.**

| Metabolic Pathway | Overlap size | Pathway size | Enrichment *P* | Model *P* |
| --- | --- | --- | --- | --- |
| 24AL vs 12AL |  |  |  |  |
| Glycerophospholipid metabolism | 7 | 19 | 0.06 | <0.01 |
| Linoleate metabolism | 6 | 15 | 0.05 | <0.01 |
| Vitamin A (retinol) metabolism | 5 | 18 | 0.26 | 0.01 |
| Glycosphingolipid metabolism | 5 | 19 | 0.30 | 0.01 |
| Sialic acid metabolism | 4 | 17 | 0.43 | 0.02 |
| Drug metabolism - cytochrome P450 | 4 | 18 | 0.48 | 0.03 |
| Omega-3 fatty acid metabolism | 2 | 6 | 0.33 | 0.03 |
| Selenoamino acid metabolism | 2 | 7 | 0.41 | 0.04 |
| C21-steroid hormone biosynthesis and metabolism | 6 | 34 | 0.68 | 0.05 |
| 10CR vs 12AL |  |  |  |  |
| Xenobiotics metabolism | 5 | 22 | 0.07 | <0.01 |
| Pyrimidine metabolism | 4 | 18 | 0.11 | 0.01 |
| Aminosugars metabolism | 3 | 13 | 0.14 | 0.01 |
| Saturated fatty acids beta-oxidation | 2 | 8 | 0.20 | 0.03 |
| Tyrosine metabolism | 4 | 29 | 0.36 | 0.03 |
| Aspartate and asparagine metabolism | 3 | 19 | 0.32 | 0.04 |
| Starch and Sucrose Metabolism | 2 | 9 | 0.24 | 0.04 |
| 20CR vs 12AL |  |  |  |  |
| Vitamin E metabolism | 11 | 23 | 0.10 | <0.01 |
| Omega-3 fatty acid metabolism | 4 | 6 | 0.10 | 0.01 |
| Valine, leucine and isoleucine degradation | 6 | 12 | 0.18 | 0.01 |
| Carnitine shuttle | 8 | 19 | 0.28 | 0.02 |
| Butanoate metabolism | 4 | 8 | 0.26 | 0.03 |
| Vitamin D3 (cholecalciferol) metabolism | 4 | 8 | 0.26 | 0.03 |
| CoA Catabolism | 2 | 2 | 0.11 | 0.03 |
| Pyrimidine metabolism | 7 | 18 | 0.39 | 0.04 |
| Fatty acid activation | 6 | 15 | 0.38 | 0.04 |
| 30CR vs 12AL |  |  |  |  |
| Vitamin E metabolism | 12 | 23 | 0.01 | <0.01 |
| Squalene and cholesterol biosynthesis | 10 | 23 | 0.09 | <0.01 |
| Tryptophan metabolism | 10 | 24 | 0.12 | 0.01 |
| Omega-3 fatty acid metabolism | 4 | 6 | 0.06 | 0.01 |
| Linoleate metabolism | 7 | 15 | 0.11 | 0.01 |
| 40CR vs 12AL |  |  |  |  |
| Linoleate metabolism | 10 | 15 | 0.03 | <0.01 |
| Tyrosine metabolism | 16 | 29 | 0.06 | <0.01 |
| Vitamin D3 (cholecalciferol) metabolism | 6 | 8 | 0.05 | <0.01 |
| Carnitine shuttle | 11 | 19 | 0.08 | <0.01 |
| Aspartate and asparagine metabolism | 11 | 19 | 0.08 | <0.01 |
| Tryptophan metabolism | 13 | 24 | 0.11 | <0.01 |
| Vitamin E metabolism | 12 | 23 | 0.16 | <0.01 |
| Glutathione Metabolism | 3 | 3 | 0.06 | <0.01 |
| Polyunsaturated fatty acid biosynthesis | 3 | 3 | 0.06 | <0.01 |
| Hexose phosphorylation | 6 | 10 | 0.16 | <0.01 |
| Fatty acid activation | 8 | 15 | 0.21 | <0.01 |
| Saturated fatty acids beta-oxidation | 5 | 8 | 0.17 | 0.01 |
| Omega-3 fatty acid metabolism | 4 | 6 | 0.18 | 0.01 |
| Lysine metabolism | 5 | 9 | 0.26 | 0.01 |
| Vitamin B6 (pyridoxine) metabolism | 3 | 4 | 0.18 | 0.01 |
| Glycosphingolipid biosynthesis - globoseries | 3 | 4 | 0.18 | 0.01 |
| N-Glycan biosynthesis | 3 | 4 | 0.18 | 0.01 |
| Fatty Acid Metabolism | 4 | 7 | 0.29 | 0.02 |
| Glycine, serine, alanine and threonine metabolism | 7 | 15 | 0.38 | 0.02 |
| Bile acid biosynthesis | 15 | 36 | 0.47 | 0.02 |
| Pentose and Glucuronate Interconversions | 2 | 2 | 0.16 | 0.03 |
| R Group Synthesis | 2 | 2 | 0.16 | 0.03 |
| De novo fatty acid biosynthesis | 6 | 13 | 0.42 | 0.03 |
| Butanoate metabolism | 4 | 8 | 0.40 | 0.04 |
| Glycerophospholipid metabolism | 8 | 19 | 0.51 | 0.04 |
| Glycosphingolipid metabolism | 8 | 19 | 0.51 | 0.04 |
| Urea cycle/amino group metabolism | 8 | 19 | 0.51 | 0.04 |

## **Table 5: Amino acid pathways altered across all CR levels. Data from mummichog pathway analysis.**

| CR level | Pathway | Features increased | Features decreased | P-value |
| --- | --- | --- | --- | --- |
| 10CR | Tyrosine metabolism | 1 | 3 | 0.034 |
| 10CR | Aspartate and asparagine metabolism | 1 | 2 | 0.036 |
| 20CR | Valine, leucine and isoleucine degradation | 2 | 4 | 0.011 |
| 30CR | Tryptophan metabolism | 5 | 5 | 0.005 |
| 40CR | Tyrosine metabolism | 6 | 12 | <0.001 |
| 40CR | Aspartate and asparagine metabolism | 3 | 10 | 0.001 |
| 40CR | Tryptophan metabolism | 5 | 11 | 0.001 |
| 40CR | Lysine metabolism | 2 | 4 | 0.011 |
| 40CR | Glycine, serine, alanine and threonine metabolism | 2 | 7 | 0.019 |

## **Table 6: Fatty acid pathways altered across all CR levels and 24AL compared to 12AL. Data from mummichog pathway analysis.**

| CR level | Pathway | Features increased | Features decreased | P-value |
| --- | --- | --- | --- | --- |
| 10CR | Saturated fatty acids beta-oxidation | 2 | 0 | 0.033 |
| 20CR | Carnitine shuttle | 7 | 1 | 0.019 |
| 20CR | Fatty acid activation | 5 | 1 | 0.043 |
| 20CR | Omega-3 fatty acid metabolism | 5 | 0 | 0.009 |
| 30CR | Squalene and cholesterol biosynthesis | 6 | 4 | 0.004 |
| 30CR | Linoleate metabolism | 6 | 1 | 0.006 |
| 30CR | Omega-3 fatty acid metabolism | 6 | 0 | 0.006 |
| 40CR | Carnitine shuttle | 6 | 0 | 0.001 |
| 40CR | Linoleate metabolism | 8 | 2 | 0.001 |
| 40CR | Polyunsaturated fatty acid biosynthesis | 3 | 0 | 0.004 |
| 40CR | Fatty acid activation | 7 | 1 | 0.005 |
| 40CR | Saturated fatty acids beta-oxidation | 5 | 3 | 0.005 |
| 40CR | Omega-3 fatty acid metabolism | 6 | 0 | 0.007 |
| 40CR | Glycosphingolipid biosynthesis - globoseries | 1 | 2 | 0.012 |
| 40CR | Fatty Acid Metabolism | 3 | 2 | 0.017 |
| 40CR | Bile acid biosynthesis | 14 | 1 | 0.021 |
| 40CR | De novo fatty acid biosynthesis | 6 | 0 | 0.027 |
| 40CR | Glycerophospholipid metabolism | 7 | 2 | 0.037 |
| 40CR | Glycosphingolipid metabolism | 6 | 2 | 0.037 |
| 24AL | Omega-3 fatty acid metabolism | 0 | 4 | 0.028 |
| 24AL | Glycerophospholipid metabolism | 2 | 7 | 0.001 |
| 24AL | Linoleate metabolism | 1 | 5 | 0.001 |
| 24AL | Glycosphingolipid metabolism | 1 | 4 | 0.008 |

## **Table 7: Correlation of amino acids (log2 fold change) with the fat free mass (g) and fasting glucose (mmol/l). Benjamini-Hochberg adjusted P-value. P-values <0.05 in bold.**

| Metabolite | R Fat free mass (g) | Adjusted *P* | R Fat mass (g) | Adjusted *P* | R Fasting glucose (mmol/l) | Adjusted *P* |
| --- | --- | --- | --- | --- | --- | --- |
| L-leucine | **0.716** | **<0.001** | **0.468** | **0.008** | **0.544** | **<0.001** |
| L-glutamate | **0.811** | **<0.001** | **0.597** | **<0.001** | - | - |
| L-tyrosine | **0.698** | **<0.001** | **0.384** | **0.028** | - | - |
| L-phenylalanine | **0.675** | **<0.001** | **0.342** | **0.047** | - | - |
| S-adenosyl-L-methionine | **-0.563** | **<0.001** | **-0.368** | **0.033** | - | - |
| L-tryptophan | 0.126 | 0.615 | -0.059 | 0.814 | - | - |
| L-valine | **0.542** | **<0.001** | **0.307** | **0.076** | **0.193** | **0.198** |
| L-cysteine | **0.359** | **0.03** | **0.392** | **0.028** | - | - |
| L-glutamine | 0.013 | 0.97 | -0.037 | 0.815 | - | - |
| L-serine | -0.062 | 0.798 | -0.124 | 0.641 | - | - |
| L-methionine | **0.545** | **<0.001** | **0.474** | **0.008** | - | - |
| L-proline | **0.427** | **0.008** | **0.428** | **0.016** | - | - |
| Cysteine-S-sulphate | 0.006 | 0.97 | -0.099 | 0.718 | - | - |
| N-acetyl-L-glutamate | 0.105 | 0.629 | -0.045 | 0.815 | - | - |
| L-arginine | -0.118 | 0.615 | -0.060 | 0.814 | - | - |

## **Table 8: Correlation of amino acids (log2 fold change) with plasma measured hormones, leptin insulin and TNF-α levels and visceral fat mass (g) after 12 weeks of CR. Benjamini-Hochberg adjusted P-value. P-values <0.05 in bold.**

| Metabolite | *R* Leptin | Adjusted *P* | *R* TNF | Adjusted *P* | *R* Insulin | Adjusted *P* | *R* Visceral fat mass (g) | Adjusted *P* |
| --- | --- | --- | --- | --- | --- | --- | --- | --- |
| L-leucine | 0.40 | **0.05** | 0.21 | 0.26 | 0.43 | **0.02** | **0.52** | **<0.01** |
| L-glutamate | 0.50 | **0.01** | 0.43 | **0.04** | 0.52 | **0.01** | **0.66** | **<0.01** |
| L-tyrosine | 0.33 | 0.08 | 0.36 | 0.08 | 0.28 | 0.11 | **0.56** | **<0.01** |
| L-phenylalanine | 0.33 | 0.08 | 0.25 | 0.20 | 0.27 | 0.13 | **0.47** | **<0.01** |
| S-Adenosyl-L-methionine | -0.38 | 0.06 | -0.53 | **0.01** | -0.47 | **0.01** | **-0.48** | **<0.01** |
| L-valine | 0.24 | 0.17 | 0.24 | 0.20 | 0.34 | 0.06 | **0.41** | **0.01** |
| L-cystine | 0.47 | **0.02** | 0.38 | 0.07 | 0.46 | **0.01** | **0.41** | **0.01** |
| L-methionine | 0.28 | 0.12 | 0.16 | 0.36 | 0.39 | **0.04** | **0.48** | **<0.01** |
| L-proline | 0.31 | 0.09 | 0.32 | 0.11 | 0.34 | 0.06 | **0.36** | **0.03** |

## **Table 9: Correlation of amino acids (log2 fold change) with the hypothalamic transcripts of cholinergic receptor levels after 12 weeks of CR. Benjamini-Hochberg adjusted P-value. P-values <0.05 in bold.**

| Metabolite | *R Adrbk2* |  | Adjusted *P* | *R Drd5* | Adjusted *P* |
| --- | --- | --- | --- | --- | --- |
| L-tyrosine | -0.36 |  | 0.13 | -0.47 | **0.03** |
| L-phenylalanine | 0.64 |  | **0.03** | -0.21 | 0.93 |
| L-tryptophan | 0.23 |  | **0.01** | 0.12 | 0.91 |
| Dopamine-3-sulfate | -0.20 |  | 0.67 | -0.48 | 0.053 |

## **Table 10: Correlation of vitamins and vitamin derivatives with organs of the alimentary tract that were invested in after 3 months of CR. Benjamini-Hochberg adjusted P-value. P-values <0.05 in bold. Vitamin group in brackets.**

| Metabolite | Stomach | Adjusted *P* | Colon | Adjusted *P* |
| --- | --- | --- | --- | --- |
| reduced riboflavin (B2) | **0.564** | **<0.001** | **0.510** | **0.002** |
| 11'-carboxy-γ-tocotrienol (E) | **0.367** | **0.048** | **0.376** | **0.032** |
| pantothenate (B5) | **0.420** | **0.020** | 0.266 | 0.132 |
| γ-tocotrienol (E) | **0.556** | **<0.001** | **0.344** | **0.039** |
| 4-oxo-9-cis-retinoate (A) | 0.246 | 0.198 | 0.208 | 0.241 |
| 13-cis-retinoate (A) | 0.328 | 0.083 | **0.360** | **0.037** |
| 13'-hydroxy-α-tocotrienol (E) | 0.294 | 0.108 | **0.348** | **0.039** |
| α-tocotrienol (E) | 0.135 | 0.496 | **0.439** | **0.009** |
| 13'-carboxy-γ-tocopherol (E) | 0.110 | 0.534 | **0.463** | **0.006** |
| 13'-carboxy-γ-tocotrienol (E) | 0.152 | 0.457 | -0.082 | 0.630 |
| retinoic acid (A) | 0.118 | 0.534 | 0.019 | 0.900 |
| vitamin K | 0.305 | 0.104 | 0.256 | 0.137 |
| Retinal (A) | -0.066 | 0.707 | -0.160 | 0.385 |
| riboflavin (B2) | -0.176 | 0.389 | -0.145 | 0.415 |
| 11'-carboxy-α-tocotrienol (E) | 0.215 | 0.267 | **0.544** | **0.002** |
| 13'-carboxy-α-tocotrienol (E) | 0.015 | 0.919 | 0.102 | 0.573 |

## **Table 11: Correlation of vitamins and vitamin derivatives with measures of oxidative stress and leptin. DROMS=reactive oxidative metabolites. Benjamini-Hochberg adjusted P-value. P-values <0.05 in bold. Vitamin group in brackets.**

| Metabolite | *R* Catalase | Adjusted *P* | *R* DROMS | Adjusted *P* | *R* Leptin | Adjusted *P* |
| --- | --- | --- | --- | --- | --- | --- |
| reduced riboflavin (B2) | **-0.386** | **0.026** | **0.642** | **<0.001** | **-** | **-** |
| 11'-carboxy-γ-tocotrienol (E) | **-0.433** | **0.014** | **0.473** | **0.005** | **-0.612** | **0.033** |
| pantothenate (B5) | **-0.423** | **0.014** | **0.505** | **0.003** | **-** | **-** |
| γ-tocotrienol (E) | **-0.496** | **0.004** | **0.731** | **<0.001** | **-0.601** | **0.021** |
| 4-oxo-9-cis-retinoate (A) | -0.248 | 0.247 | **0.434** | **0.010** | **-** | **-** |
| 13-cis-retinoate (A) | **-0.515** | **0.004** | **0.469** | **0.005** | **-** | **-** |
| 13'-hydroxy-α-tocotrienol (E) | -0.183 | 0.398 | **0.555** | **0.001** | **-** | **-** |
| α-tocotrienol (E) | -0.233 | 0.247 | 0.258 | 0.158 | **-** | **-** |
| 13'-carboxy-γ-tocopherol (E) | -0.137 | 0.484 | 0.186 | 0.336 | **-** | **-** |
| 13'-carboxy-γ-tocotrienol (E) | -0.163 | 0.406 | 0.093 | 0.605 | **-** | **-** |
| retinoic acid (A) | 0.093 | 0.633 | **0.408** | **0.016** | **-** | **-** |
| vitamin K | -0.230 | 0.247 | **0.501** | **0.003** | **-** | **-** |
| Retinal (A) | 0.169 | 0.406 | 0.142 | 0.471 | **-** | **-** |
| riboflavin (B2) | -0.034 | 0.822 | -0.033 | 0.840 | **-** | **-** |
| 11'-carboxy-α-tocotrienol (E) | 0.076 | 0.659 | **0.347** | **0.045** | **-** | **-** |
| 13'-carboxy-α-tocotrienol (E) | -0.090 | 0.633 | -0.124 | 0.509 | **-** | **-** |
| 13’-hydroxy-γ-tocotrienol (E) | - | - | - | - | **-0.464** | **0.030** |

## **Table 12: Pathways potentially associated with lifespan from mummichog.**

| Pathway | Overlap size | Pathway size | P-value (raw) | P-value | Association with lifespan |
| --- | --- | --- | --- | --- | --- |
| Linoleate metabolism | 12 | 15 | 0.00 | <0.01 | positive |
| Vitamin D3 (cholecalciferol) metabolism | 7 | 8 | 0.00 | <0.01 | positive |
| Tyrosine metabolism | 16 | 29 | 0.01 | <0.01 | negative |
| Vitamin E metabolism | 11 | 23 | 0.11 | <0.01 | positive |
| Tryptophan metabolism | 11 | 24 | 0.15 | <0.01 | negative |
| Glutathione Metabolism | 3 | 3 | 0.04 | <0.01 | negative |
| Omega-3 fatty acid metabolism | 4 | 6 | 0.10 | <0.01 | positive |
| Lysine metabolism | 5 | 9 | 0.15 | <0.01 | negative |
| Glycine, serine, alanine and threonine metabolism | 7 | 15 | 0.21 | <0.01 | negative |
| Fatty acid activation | 7 | 15 | 0.21 | <0.01 | positive |
| Fatty Acid Metabolism | 4 | 7 | 0.18 | <0.01 | positive |
| Histidine metabolism | 4 | 7 | 0.18 | <0.01 | negative |
| Putative anti-Inflammatory metabolites formation from EPA | 7 | 16 | 0.28 | 0.01 | positive |
| Glycerophospholipid metabolism | 8 | 19 | 0.29 | 0.01 | positive |
| Aspartate and asparagine metabolism | 8 | 19 | 0.29 | 0.01 | negative |
| Squalene and cholesterol biosynthesis | 9 | 23 | 0.37 | 0.01 | positive |
| Butanoate metabolism | 4 | 8 | 0.27 | 0.01 | negative |
| Saturated fatty acids beta-oxidation | 4 | 8 | 0.27 | 0.01 | negative |
| Bile acid biosynthesis | 13 | 36 | 0.45 | 0.01 | positive |
| Valine, leucine and isoleucine degradation | 5 | 12 | 0.38 | 0.01 | negative |
| De novo fatty acid biosynthesis | 5 | 13 | 0.46 | 0.02 | positive |
| Polyunsaturated fatty acid biosynthesis | 2 | 3 | 0.27 | 0.03 | positive |
| Beta-Alanine metabolism | 2 | 3 | 0.27 | 0.03 | negative |
| Hexose phosphorylation | 4 | 10 | 0.46 | 0.03 | negative |
| Fructose and mannose metabolism | 4 | 11 | 0.54 | 0.04 | negative |
| Leukotriene metabolism | 9 | 28 | 0.65 | 0.04 | positive |

## **Table 13: Pathways potentially associated with lifespan associated pathways from IPA**

| Ingenuity Canonical Pathways | P-value | Ratio | Association with lifespan |
| --- | --- | --- | --- |
| Tyrosine Biosynthesis IV | <0.01 | 0.75 | negative |
| Phenylalanine Degradation I (Aerobic) | <0.01 | 0.429 | negative |
| (S)-reticuline Biosynthesis II | <0.01 | 0.333 | negative |
| Catecholamine Biosynthesis | <0.01 | 0.273 | negative |

## **Table 14: Plasma metabolites and hypothalamus mRNA transcripts correlated with level of CR.**

| Molecule/mRNA transcript | *R* Treatment level | Adjusted *P* |  |
| --- | --- | --- | --- |
| Dopamine-3-O-sulfate | -0.609 | <0.001 | |
| S-adenosyl-L-methionine | 0.483 | 0.003 | |
| Linoleate | 0.521 | 0.004 | |
| gamma-Linolenic acid | 0.514 | 0.001 | |
| 12-HETE-Gly | 0.736 | <0.001 | |
| 13(S)-HPOT | 0.866 | <0.001 | |
| Sphinganine | 0.523 | <0.001 | |
| L-carnitine | -0.507 | <0.001 | |
| Heptadecanoyl carnitine | 0.480 | 0.003 | |
| Tetradecanoyl carnitine | 0.507 | 0.004 | |
| O-acetylcarnitine | 0.648 | 0.004 | |
| L-acetylcarnitine | 0.676 | 0.004 | |
| Stearidonyl carnitine | -0.359 | 0.025 | |
| 7α-hydroxycholesterol | 0.491 | <0.001 | |
| 5β-Cholestane-3α,7α-diol | 0.447 | <0.001 | |
| 5β-Cholestane-3α,7α,26-triol | 0.661 | <0.001 | |
| 5β-cyprinolsulphate | 0.566 | 0.003 | |
| 7α-Hydroxy-3-oxo-4-cholestenoate | 0.515 | 0.020 | |
| Taurine | -0.422 | 0.040 | |
| *Drd5* | 0.499 | 0.005 | |

**Table 15: Correlations between the average final two weeks of body temperature (Tb), food anticipatory activity (FAA) and total physical activity (PA) with the metabolite anandamide.**

|  | *R* anandamide | *P* |
| --- | --- | --- |
| Tb | -0.480 | 0.001 |
| FAA | 0.541 | <0.001 |
| PA | 0.031 | 0.841 |
